# Clinical Interpretation of Serum Troponin in the Era of High-Sensitivity Testing

**DOI:** 10.3390/diagnostics14050503

**Published:** 2024-02-26

**Authors:** Marah Maayah, Scott Grubman, Stephanie Allen, Zachary Ye, Dae Yong Park, Evangelia Vemmou, Ilhan Gokhan, Wendy W. Sun, Stephen Possick, Jennifer M. Kwan, Parul U. Gandhi, Jiun-Ruey Hu

**Affiliations:** 1 Yale School of Medicine, Yale University, New Haven, CT 06520, USAilhan.gokhan@yale.edu (I.G.); 2 Department of Internal Medicine, Yale School of Medicine, Yale University, New Haven, CT 06520, USAevangelia.vemmou@yale.edu (E.V.); 3 Department of Internal Medicine, Temple University Medical Center, Philadelphia, PA 19140, USA; 4 Department of Internal Medicine, Cook County Hospital, Chicago, IL 60612, USA; 5 Department of Emergency Medicine, Yale School of Medicine, Yale University, New Haven, CT 06520, USA; wendy.w.sun@yale.edu; 6 Section of Cardiovascular Medicine, Yale School of Medicine, Yale University, New Haven, CT 06520, USA; 7 Department of Cardiology, Veterans Affairs Connecticut Health Care System, West Haven, CT 06516, USA

**Keywords:** troponin, myocardial injury, myocardial ischemia, myocardial infarction, supply–demand mismatch

## Abstract

Cardiac troponin (Tn) plays a central role in the evaluation of patients with angina presenting with acute coronary syndrome. The advent of high-sensitivity assays has improved the analytic sensitivity and precision of serum Tn measurement, but this advancement has come at the cost of poorer specificity. The role of clinical judgment is of heightened importance because, more so than ever, the interpretation of serum Tn elevation hinges on the careful integration of findings from electrocardiographic, echocardiographic, physical exam, interview, and other imaging and laboratory data to formulate a weighted differential diagnosis. A thorough understanding of the epidemiology, mechanisms, and prognostic implications of Tn elevations in each cardiac and non-cardiac etiology allows the clinician to better distinguish between presentations of myocardial ischemia and myocardial injury—an important discernment to make, as the treatment of acute coronary syndrome is vastly different from the workup and management of myocardial injury and should be directed at the underlying cause.

## 1. Introduction

The role of serum troponin (Tn) testing in the evaluation of patients with acute coronary syndrome (ACS) has evolved as advances have been made in the analytic sensitivity and precision of serum Tn testing. While the increased sensitivity of newer generations of serum Tn assays has the potential to improve the rule-out of ACS, this comes at the cost of poorer specificity. Mild elevations in serum Tn, when detected in otherwise asymptomatic patients, can lead to unnecessary follow-up testing, preventable radiation exposure, misdiagnosis, and financial burden to patients who do not have anginal symptoms. In one study of 44,448 patients, serum Tn testing was performed in 47% of emergency department patients admitted to the inpatient setting, 35% of whom had no anginal symptoms [1]. Elevations in serum Tn inform the clinician about the presence of myocardial injury, but not the mechanism. The interpretation of abnormal Tn levels is both a science and an art that hinges on a careful consideration of findings from electrocardiographic, echocardiographic, physical exam, interview, and other imaging and laboratory data to formulate a weighted differential diagnosis. In the accompanying biochemistry companion to this review [2], we highlight the molecular structure of the Tn complex, describe its isoforms, and explore differences between the generations of Tn assays, including the characteristics of conventional vs. high-sensitivity Tn (hs-Tn) testing. In this review, focused on the clinical interpretation of serum Tn elevations, we explore the evolution of terminology surrounding myocardial injury, distinguishing between myocardial injury, myocardial ischemia, and myocardial infarction. We also review the spectrum of cardiac and non-cardiac etiologies of abnormal serum Tn, as well as the epidemiology, mechanisms, and prognostic implications of each condition. Given the wealth of resources focused on ACS in existence, the present review will focus on non-ACS etiologies of Tn elevation.

## 2. Distinguishing between Myocardial Injury, Myocardial Ischemia, and Myocardial Infarction

### 2.1. Historical Evolution of Terminology for Myocardial Injury

The meaning of the term “myocardial infarction” (MI) varied widely until the World Health Organization first began to standardize heart disease nomenclature in the 1950s–1970s, largely using electrocardiogram (ECG)-based criteria [3]. Over the next several decades, joint task forces from the American College of Cardiology, American Heart Association, European Society of Cardiology, and World Heart Federation issued four successive iterations of the Universal Definition of Myocardial Infarction (UDMI) Consensus Document. As cardiac biomarker assays improved, the first UDMI in 2000 adopted the concept of using biomarkers such as serum troponin T and I (TnT/I) as a core component in the diagnosis of MI [4]. The second UDMI in 2007 introduced the five categories of MI and introduced the concept of troponin (Tn) rise and/or fall with at least one value above the 99th percentile upper reference limit (URL) [5]. The third UDMI in 2012 added criteria for the diagnosis of MI in the setting of left bundle branch block and paced rhythms and introduced an emphasis on incorporating imaging findings and the clinical presentation into the interpretation of Tn elevations [6]. The fourth and most recent UDMI published in 2018 issued updated definitions to accommodate the increased use of hs-Tn, discussed the implications of chronic elevations in Tn, and further clarified distinctions between the separate yet interrelated entities of myocardial injury, myocardial ischemia, and myocardial infarction [5].

We will begin with the current definition of these terms. There remains wide variation and inconsistency in the terminology used by practicing clinicians to describe clinical scenarios involving elevated Tn. A study of discharge summaries of patients discharged from the medical service at three Rhode Island hospitals found that 19 distinct terms were used to describe the patients’ Tn values, many of which were imprecise (e.g., “troponin bump”, “troponin leak”) or deprecated (e.g., “type 2 NSTEMI” instead of “type 2 MI” or “NSTEMI”) [7]. This suggests that greater efforts are needed to raise awareness of the terminology and paradigm espoused in the fourth UDMI, more than five years after its publication.

### 2.2. Myocardial Injury

**Figure 1:**
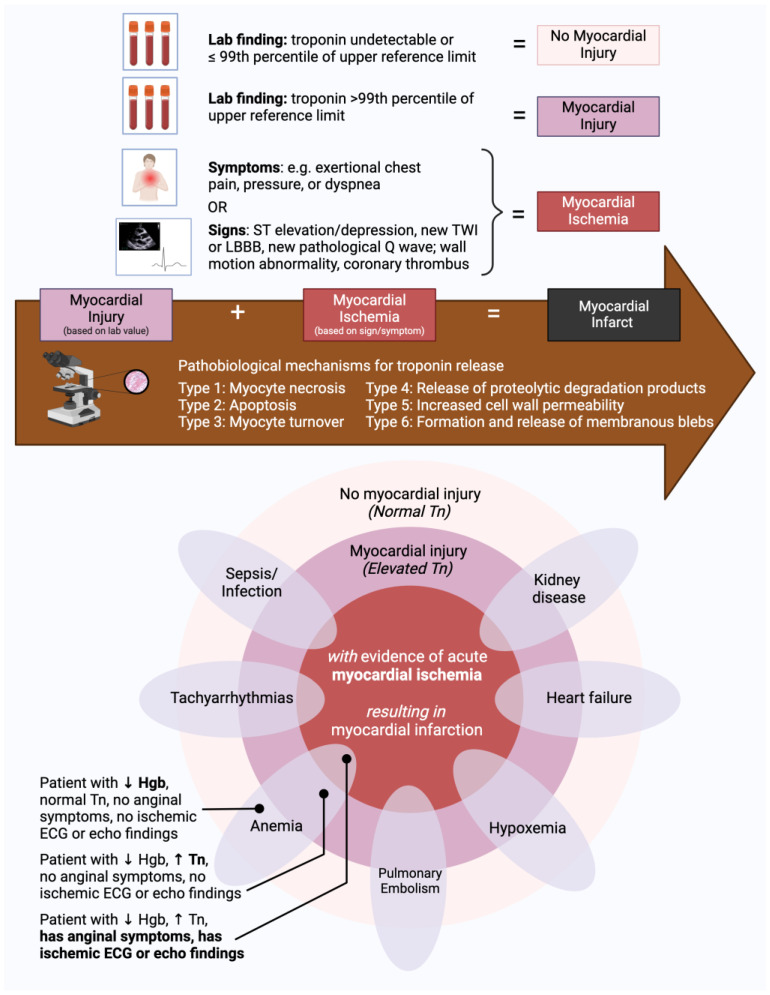
(**Top**) The relationship between myocardial injury, myocardial ischemia, and myocardial infarction. Myocardial injury refers to any patient with a Tn level above the 99th percentile URL, regardless of the underlying cause. The diagnosis of myocardial ischemia requires at least one of the following: clinical ischemic symptoms, new ischemic changes on ECG, new pathologic Q waves, a new loss of viable myocardium or wall motion abnormalities in an ischemic pattern, or angiographic or autopsy evidence of an acute coronary thrombus. Myocardial infarction is defined by the presence of both acute myocardial injury and evidence of myocardial ischemia. This process can occur via a number of pathobiological mechanisms, most commonly involving myocyte necrosis and/or apoptosis. (**Bottom**) Each disease entity (e.g., anemia) exists on a spectrum, in which it can present in isolation with no myocardial injury, with myocardial injury, or with acute myocardial ischemia leading to myocardial infarction. For example, a patient with a drop in hemoglobin, normal Tn, and no anginal symptoms, ischemic ECG changes, or echo findings, has no myocardial injury. A patient with a drop in hemoglobin, elevation in Tn, but no anginal symptoms, ischemic changes, or echo findings, has myocardial injury without myocardial ischemia. Lastly, a patient with a drop in hemoglobin, elevation in Tn, and either anginal symptoms, ischemic changes, or echo findings, has myocardial injury with myocardial ischemia, resulting in myocardial infarction. Abbreviations: ECG: electrocardiogram; Hgb: hemoglobin; LBBB: left bundle branch block; Tn: troponin; TWI: T-wave inversion. Original figure designed in BioRender.

**Figure 2:**
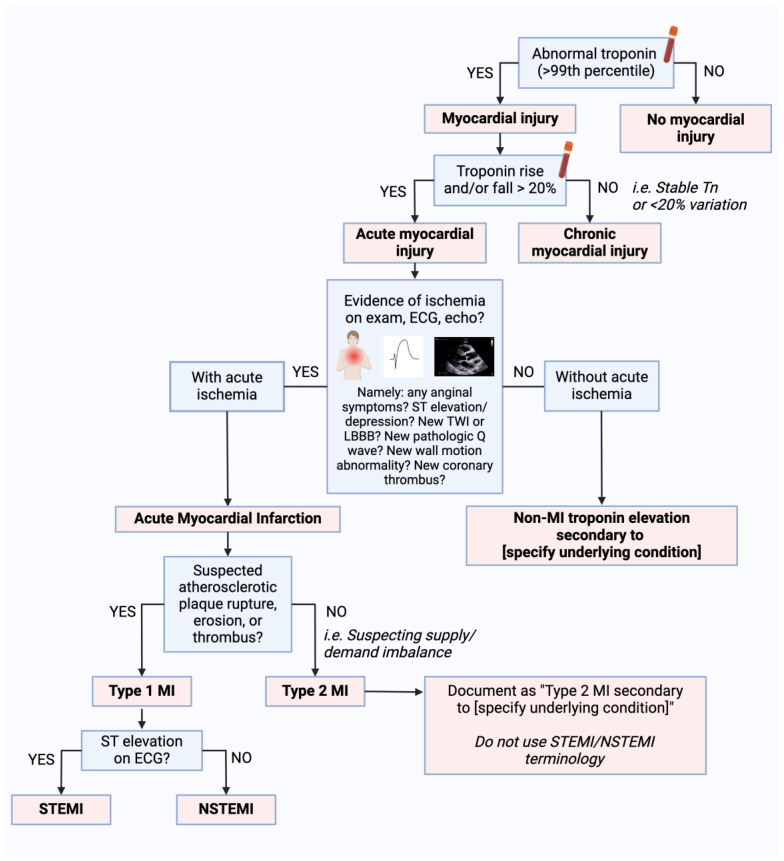
Contemporary framework for interpreting myocardial injury, myocardial ischemia, and myocardial infarction. Original figure designed in BioRender.

The term “myocardial injury” applies to any patient with a conventional or hs-Tn value above the 99th percentile URL, regardless of the underlying cause (Figure 1) [5]. Myocardial injury can be further categorized as acute or chronic [8]. Acute myocardial injury is used to describe a dynamic rise or fall in conventional or hs-Tn values exceeding typical biological or analytical variation [9]. While a consensus on the specific threshold for acute injury remains elusive, it has been proposed that an increase greater than the reference change value be considered acute for TnT or TnI if the initial value is <99th percentile URL [9]. When the patient’s baseline Tn is >99th percentile URL, Tn increases ≥ 50% of the 99th percentile URL or changes > 20% of the baseline Tn are considered acute (Figure 2) [10]. Chronic myocardial injury is characterized by persistently elevated Tn values > 99th percentile URL not meeting the above criteria [10]. There is enthusiasm for defining acute injury based on absolute “delta” changes in hs-Tn instead of the traditional 20% increase, but these delta thresholds are manufacturer-specific, and research is ongoing to define the optimal delta threshold in each setting of care [11,12]. There are many known etiologies of acute and chronic myocardial injury, including both intrinsic cardiac and non-cardiac conditions, to be discussed below.

It is crucial for clinicians to differentiate between nonischemic myocardial injury, which may be associated with conditions such as renal failure or sepsis, and myocardial injury in the setting of acute myocardial ischemia with or without atherosclerotic plaque disruption. Clinicians should avoid reflexively equating elevated Tn levels with myocardial infarction solely on the basis of an elevated biomarker. If ischemic ECG changes and/or symptoms are lacking, a diagnosis of “non-MI Tn elevation due to (an underlying cause)” would be more accurate. At times, there may be an overlap between myocardial ischemic and nonischemic conditions associated with increased Tn values. In these situations, it may be difficult to discriminate between the mechanisms of myocardial injury. It is important to document all possible contributors and their relative contributions. The terms “troponinemia”, “troponin leak”, “troponin bump”, and “troponinitis” should not be used in official documentation in the electronic medical record [13].

### 2.3. Myocardial Ischemia

Myocardial ischemia is defined as a mismatch between myocardial oxygen supply and metabolic demand [14]. The clinical diagnosis of myocardial ischemia requires at least one of the following: clinical ischemic symptoms, new ischemic changes on ECG, new pathologic Q waves, a new loss of viable myocardium or wall motion abnormalities in an ischemic pattern, or angiographic or autopsy evidence of an acute coronary thrombus [5]. Classically, myocardial ischemia is the result of coronary luminal narrowing secondary to atherosclerosis [8]. Sufficiently high-grade stenosis in the absence of adequate collateralization can lead to ischemic physiology with exertion, often referred to as “chronic coronary artery disease (CAD)”, “chronic coronary syndrome”, “chronic coronary disease”, or “stable ischemic heart disease” (SIHD) [15,16]. SIHD classically presents as stable angina, although it can also manifest as chest pressure, jaw discomfort, arm discomfort, dyspnea, and epigastric discomfort [17]. There is also increasing recognition that processes such as epicardial coronary or microvascular dysfunction can also play a role in the development of myocardial ischemia, with or without concurrent obstructive atherosclerotic disease [18]. More rarely, ischemic damage can result from alternative pathologies, such as coronary dissection, arteritis, myocardial bridging, thromboembolism, or vasospasm [19].

### 2.4. Myocardial Infarction

**Figure 3:**
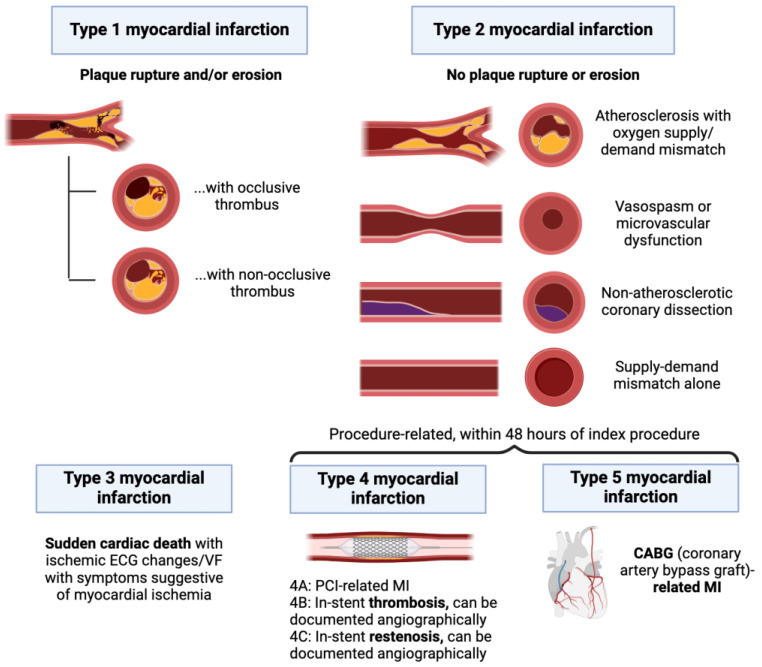
Pathophysiology of type 1, 2, 3, 4A, 4B, 4C and 5 myocardial infarctions (MIs). Abbreviations: CABG: coronary artery bypass grafting; ECG: electrocardiogram; PCI: percutaneous coronary intervention; VF: ventricular fibrillation. Original figure designed in BioRender.

Myocardial infarction (MI) is defined by the presence of both acute myocardial injury and evidence of myocardial ischemia [5]. Pathologically, MI is typically characterized by myocardial cell death, with necrosis progressing from the subendocardium to the subepicardium over the course of several hours [20]. Diagnosing MI requires careful consideration of findings from a patient’s history, physical exam, labs, ECG, and imaging. There are five types of MI delineated under current joint society guidelines (Figure 3) [5].

Type 1 MI is caused by atherosclerotic plaque rupture or ulceration and resultant intraluminal thrombosis, which may also be complicated by hemorrhage into the plaque [21]. The appearance of new ST-segment elevations in ≥2 contiguous leads or the appearance of a new bundle branch block with ischemic repolarization patterns indicates an ST-elevation MI (STEMI), whereas the presence of a Tn elevation in the absence of these ECG changes indicates a non-ST-elevation MI (NSTEMI). The umbrella of ACS spans STEMI, NSTEMI, and unstable angina [22]. While the increasing sensitivity of Tn assays has reclassified many cases of unstable angina to NSTEMI, unstable angina has not yet disappeared [23]. The terms “STEMI” and “NSTEMI” should only be used when referring to type 1 MI and should be differentiated from type 2 MI and non-MI Tn elevation due to a non-cardiac cause. For instance, “type 2 NSTEMI” is a misnomer.

Type 2 MI is characterized by a mismatch between myocardial oxygen supply and demand; however, unlike patients with type 1 MI, patients with type 2 MI are not experiencing an acute plaque rupture event. Diagnosis requires evidence of myocardial injury alongside at least one of the following findings: anginal symptoms, ischemic ECG changes, or imaging suggestive of a new loss of viable myocardium [5]. Angiography excluding acute plaque rupture is not necessary for the diagnosis of type 2 MI, although it may be otherwise clinically indicated to distinguish type 2 from type 1 MI. Some evidence suggests early cardiac magnetic resonance imaging (MRI) may also have prognostic value in type 2 MI; one study of 437 patients with MI but without coronary obstruction found that late gadolinium enhancement and abnormal T2 mapping values on cardiac MRI were both associated with an increased risk of adverse cardiac events [24]. Type 2 MI can be caused by increased demand alone, such as during episodes of severe hypertension or sustained tachyarrhythmia, or by reduced oxygen delivery alone, such as in cases of severe anemia, hypoxemia, or bradyarrhythmia. Type 2 MI may also be caused by an acute stressor in the setting of underlying coronary disease, including atherosclerosis, microvascular disease, vasospasm, dissection, or embolism [5].

Type 3, 4, and 5 MIs describe MIs that occur in patients who expire prior to biomarker confirmation, MI attributable to percutaneous coronary interventions (PCIs), and MI attributable to coronary artery bypass grafting (CABG) surgery, respectively [5]. As myocardial injury is expected after both PCI and CABG, Tn thresholds for type 4 and 5 MIs are set more stringently by the fourth UDMI at >5 or >10 times the 99th percentile URL in those with a normal baseline Tn, respectively (or at a ≥20% increase to these levels in those with an elevated baseline) [4]. Other entities, such as the Academic Research Consortium, suggest a higher threshold of ≥35 times the 99th percentile URL [25].

Corroborating Tn elevations with clinical, ECG, or imaging findings is imperative to the accurate diagnosis of type 4 or 5 MI. Myocardial injury alone is not strongly correlated with infarction in PCI or CABG patients. One study of 673 consecutive patients undergoing left main PCI found that isolated Tn elevation above the UDMI threshold for type 4 MI was not associated with a higher risk of in-hospital or long-term all-cause mortality (PMID: 36615044). Another study randomized 60 patients with normal left ventricular (LV) ejection fraction to either on- or off-pump CABG; the patients were followed with serial conventional TnI measurements and assessed for irreversible myocardial damage using contrast-enhanced cardiac magnetic resonance imaging (CMR). New irreversible myocardial damage was observed on CMR in 36% of patients in the on-pump group vs. 44% of those in the off-pump group (*p* = 0.8). While patients in both the on-pump and off-pump groups had high median area-under-the-curve (AUC) serial TnI values (182 vs. 135 μg/L; *p* = 0.02), individual AUC TnI values were found to be only moderately correlated with the mean mass of new hyperenhancement on CMR (r2 = 0.4; *p* = 0.008) [26].

### 2.5. Mechanisms of Troponin Release

The question of whether Tn release can occur in the absence of myocyte necrosis has been a subject of debate over the last few decades, as has the question of whether myocardial injury is an irreversible or reversible process. While, historically, all forms of Tn elevation were thought to result from myocyte necrosis [27], it is now understood that Tn elevation can also occur without myocyte necrosis [28]. Tn release can occur via six major mechanisms: (1) myocyte necrosis, which is the most common mechanism; (2) apoptosis, whereby caspases are activated that mediate the cleavage of structural proteins during programmed cell death; (3) myocyte cell turnover, which occurs at a low grade throughout the lifespan; (4) cellular release of proteolytic Tn degradation products in the absence of cell death or cell membrane disruption, whereby small fragments of troponin are able to pass through a cell membrane that is still intact; (5) increased cell wall permeability, which can occur via myocardial stretch or ischemia; (6) active secretion of vesicles in the absence of necrosis [29]. At a clinical level, it is not possible to definitively distinguish which increases in Tn levels are due to which mechanisms [28]. Although it is not possible to equate myocardial injury with necrosis, the pathway of myocardial injury and myocardial ischemia resulting in myocardial infarction occurs via myocyte necrosis in the majority of cases.

## 3. Clinical Interpretation of High-Sensitivity Troponin

### 3.1. Implications of High-Sensitivity Testing

As described in the biochemistry companion to this review, successive generations of Tn assays have increased the sensitivity, precision, and speed of testing [2]. The term “high-sensitivity” refers to any assay whose total imprecision (as defined by the coefficient of variance) at the 99th percentile value is ≤10% [30]. Compared with conventional (first to fourth generations) Tn assays, hs-Tn assays have higher negative predictive value for acute MI, meaning that a negative test result has a much higher certainty of truly representing the absence of acute MI. Hs-Tn assays also result in a 4% absolute increase and 20% relative increase in the detection of type 1 MI, which consequently decreases the diagnosis of unstable angina because abnormal Tn levels are more easily detected. Furthermore, hs-Tn assays result in a two-fold increase in the diagnosis of type 2 MI, reduce the “troponin-blind” interval (meaning that the time course for serial Tn checks can be shortened), and are more likely to detect circulating levels of cardiac Tn in healthy individuals without cardiovascular disease [30].

As with prior generations of Tn assays, the degree of elevation of hs-Tn can be interpreted as a quantitative marker of cardiomyocyte damage, with higher levels generally carrying a higher likelihood of MI. Elevations in hs-Tn above 5-fold the 99th percentile URL carry a >90% positive predictive value for acute type 1 MI. On the other hand, elevations in hs-Tn up to 3-fold the 99th percentile URL carry a limited (50–60%) positive predictive value for acute type 1 MI, so a thorough investigation of a broad spectrum of cardiac and non-cardiac etiologies for Tn elevation is warranted [30]. Rising or falling levels of hs-Tn are more indicative of acute cardiomyocyte damage, including acute MI, whereas stable levels of hs-Tn are more indicative of chronic cardiomyocyte damage, such as that seen in chronic kidney disease. It should also be noted that the 99th percentile URL is higher in men than the 99th percentile URL in women for both hs-TnT and hs-TnI assays [31]. The use of sex-specific thresholds for hsTn increases the diagnosis of MI in women [32] but does not necessarily improve cardiovascular outcomes [33] and remains the subject of further study. 

### 3.2. Guidelines from Specialty Societies

Several academic societies, including the American Heart Association (AHA), the American College of Cardiology (ACC), and the European Society of Cardiology (ESC), have published guidelines pertaining to the timing and interpretation of hs-Tn in the setting of suspected ACS [30,34]. 

In contrast to the AHA/ACC 2021 guidelines, which do not endorse a specific rule-in/rule-out algorithm, the ESC 2023 guidelines provide an ESC algorithm for serial hs-Tn measurements at 0 and 1 h or at 0 and 2 h to rule in and rule out NSTEMI [30,34]. The ESC 2023 guidelines recommend hs-Tn measurement at 3 h if the first two hs-Tn measurements in the 0 and 1 h algorithm are inconclusive and no alternative diagnoses explaining the condition have been made [34]. The ESC 2023 guidelines makes an additional statement with regard to the use of coronary CT angiography (CCTA).

Both the AHA/ACC 2021 and ESC 2023 guidelines recommend the use of high-sensitivity assays over non-high-sensitivity assays [30,34]. Both guidelines do not recommend the addition of older biomarkers such as CK-MB or investigational biomarkers such as copeptin in the evaluation of patients with chest pain undergoing Tn testing [30,34].

## 4. Troponin Elevation in the General Population

### 4.1. Epidemiology

As Tn assays have become more sensitive, the presence of Tn circulating in the serum has been found to be more common in the general population—including in healthy individuals—than previously thought. Of the 3557 individuals enrolled in the Dallas Heart Study to study subclinical cardiovascular disease, conventional TnT was detectable in 0.7% using a conventional assay with a detection limit of <0.01 μg/L [35]. The rate of detectable Tn is significantly higher using an hs-Tn assay, which has a lower limit of detection. For instance, in the Atherosclerosis Risk in Communities (ARIC) study, which included 9593 individuals without preexisting cardiovascular disease, hs-TnT was detectable (≥3 ng/L) in 59% and elevated (>99th percentile URL) in 7% of the study population [36]. When the ARIC study sample was narrowed only to patients with ideal cardiovascular health (including controlled cholesterol, blood pressure, and blood glucose, goal levels of physical activity, and nonsmoking status, with full criteria detailed in the 2010 American Heart Association statement) [37], the prevalence of detectable hs-TnT dropped from 59% to 44% [36]. The Scottish Family Health Study of 19,501 participants yielded similar findings, with detectable hs-TnT (>3 ng/L) found in 53.3% of individuals. This study also measured the levels of hs-TnI, with a lower limit of detection of <1.2 ng/L, and found the prevalence of detectable hs-TnI to be 74.8%, although patients with a history of cardiovascular disease were not excluded on enrollment [38].

### 4.2. Hypothesized Mechanism

The etiology of Tn elevation above the lower limit of detection but below the 99th percentile of normal in asymptomatic individuals has not been fully elucidated. Hs-Tn levels in asymptomatic individuals have positive associations with male sex, age, body mass index, systolic blood pressure (SBP), LV mass, and indices of LV systolic and diastolic function [39]. Minimal hs-Tn levels are further associated with the presence of cardiovascular risk factors, including hypertension, diabetes, and obesity [36,39]. Detectable Tn levels may also be explained by some amount of natural cardiomyocyte turnover. A study examining hearts obtained from the autopsies of 106 men and women found that while myocardial mass is preserved in women, about 1 g of mass is lost per year through the physiological loss of myocardium by necrosis and apoptosis in the normal male heart [40]. Subtle structural abnormalities, such as diastolic dysfunction, resulting from advancing age and the coexistence of cardiovascular risk factors may also contribute to Tn release in asymptomatic individuals [41].

Moreover, a study of hourly measurement of hs-TnT demonstrated that Tn levels exhibit diurnal variation, peaking in the morning (17.1 ± 2.9 ng/L at 8:30 AM), decreasing throughout the day (1.9 ± 1.6 ng/L at 8:30 PM), and then rising again overnight [42]. However, it is unclear whether these fluctuations represent a disease-specific mechanism (e.g., ischemia) or a physiological mechanism (e.g., protein turnover) associated with normal circadian rhythm patterns. Finally, extreme exercise has also been shown to cause Tn elevation in the general population, as discussed in a subsequent section.

### 4.3. Prognostic Implications

Even minimally elevated Tn in asymptomatic patients may be associated with adverse outcomes. In a meta-analysis of 28 trials measuring hs-Tn (variable assays) in 154,052 asymptomatic individuals, the levels of hs-TnI and hs-TnT at the upper limit of the normal range were predictive of cardiovascular mortality [43]. In another study including individual data from 10 prospective population-based studies and 74,738 participants, hs-TnI levels exhibited strong associations with cardiovascular mortality, first cardiovascular events, and overall mortality [44]. In each of the sections that follow, the association between minimally elevated Tn and adverse outcomes will be a recurrent theme. However, at this time, there is no consensus or guideline on how to approach mildly elevated troponins in the setting of acute illness, as clinical management does not necessarily change in the setting of acute illness. The decision on whether an ischemic evaluation is warranted should involve an individualized approach that takes into consideration all clinical data, including the nature of the acute illness and the overall patient prognosis.

## 5. Cardiovascular Etiologies of Troponin Elevation

Cardiac etiologies of Tn elevation include tachyarrhythmias, heart failure, myocarditis, pericarditis, endocarditis, hypertensive emergency, acute aortic syndromes, stress cardiomyopathy, amyloidosis, heart transplant, and cardiac procedures. The possibility of ACS should be considered alongside non-ACS causes of myocardial injury in each of these conditions, as detailed below, using clinical judgment based on findings from the history, physical exam, ECG, and imaging data, such as an echocardiogram.

### 5.1. Tachyarrhythmia

#### 5.1.1. Epidemiology

The prevalence of Tn elevation in the setting of tachyarrhythmia is generally estimated to be between 30% and 50%. In one study of 100 patients with supraventricular tachyarrhythmias (atrial fibrillation [AF], atrial flutter [AFL], and reentrant tachycardia), 44.2% of initial hs-TnT testing and 50.7% of repeat testing were positive [45]. In another study of 73 patients with tachyarrhythmia, 32.9% had abnormal conventional TnT values, and the magnitude of Tn elevation was associated with the degree of tachycardia [46]. Similarly, a study utilizing conventional TnI assays found that 37.2% of 78 patients with supraventricular tachycardia had elevated TnI [47]. On average, Tn release in the case of tachyarrhythmia is not as high as in NSTEMI [48].

#### 5.1.2. Hypothesized Mechanism

Tn elevation in the setting of tachyarrhythmia is often an indicator of myocardial injury and a supply–demand mismatch from increased energy demands rather than coronary artery obstruction or myocardial necrosis [49]. Several studies have demonstrated that many patients with Tn elevation in the setting of tachyarrhythmia have unobstructed coronary arteries on invasive coronary angiography [46,50]. In a study of 10 patients undergoing rapid atrial pacing during diagnostic angiography, rapid atrial pacing was found to increase hs-TnT levels even in patients without CAD or evidence of myocardial ischemia [51]. Another possible mechanism is a shortened duration of diastole, leading to reduced coronary perfusion and subsequent subendocardial ischemia [52].

#### 5.1.3. Prognostic Implications

The presence of elevated Tn with supraventricular tachycardia (SVT) has been associated with worse outcomes across multiple retrospective studies. For instance, one study examining 78 patients who had SVT found that even mild elevations in conventional TnI were associated with an increased risk of the composite outcome of death, myocardial infarction, or cardiovascular rehospitalization [47]. Although a more recent single-center study found no significant correlation between hs-TnI and mortality, their findings were limited by a small sample size, the presence of a nearby unaffiliated cardiology center, and an extremely low event rate (one instance of mortality from non-cardiac causes over the study period) [53]. There is increasing evidence that Tn may have higher prognostic importance in certain subsets of patients with SVT. For instance, one study demonstrated that while elevations in conventional TnI > 99th percentile URL at admission or 4 h after the onset of SVT were predictive of a composite of MI, new-onset heart failure, or sudden cardiac death, this effect was more pronounced for patients with a rate <85% of their age-predicted maximum compared to those with rates above this threshold [54]. Similarly, another retrospective trial found that over a mean follow-up period of nearly 2 years, elevated TnI on admission independently predicted a composite outcome of death, MI, or PCI only in patients with known CAD (hazard ratio = 3.3, *p* = 0.05) [55].

### 5.2. Heart Failure

#### 5.2.1. Epidemiology

Both acutely decompensated and chronic heart failure (HF) are associated with elevated TnI [56], regardless of the presence of coronary artery disease [57]. Elevated conventional Tn has been found in 15–29% of patients with chronic HF [57,58,59]. Mean conventional TnI levels are significantly higher in HF patients than in healthy controls and are negatively correlated with LV ejection fraction values in HF patients [58,59]. In a meta-analysis of 10 studies including 9289 patients with chronic HF (mostly HF with reduced ejection fraction), the median hs-TnT was 15 ng/L, 18 ng/L, and 22 ng/L in the <40%, 40–49%, and ≥50% LV ejection fraction groups, respectively [60].

#### 5.2.2. Hypothesized Mechanism

Various nonischemic and ischemic mechanisms have been proposed to explain elevated Tn levels in HF. Tn can be elevated by increased ventricular preload in the absence of coronary ischemia or coronary disease [57,61]. Some studies suggest that even brief periods of pressure overload begin to manifest biochemical and histopathologic signs of myocardial damage. For instance, a study using porcine hearts showed that 1 h of phenylephrine-induced elevations in SBP and LV end-diastolic pressure caused TnI elevations above the URL for over 24 h, as well as transient myocardial apoptosis without ischemic disturbances on imaging [62]. Pressure and volume overload increase myocardial wall stress, which can directly cause Tn release independent of ischemia [56,63,64]. It has also been hypothesized that the stimulation of stretch-responsive integrins in the overloaded state may result in Tn release [65].

Tn elevation in chronic HF may also be linked to ventricular remodeling and progressive myocyte loss from necrosis, apoptosis, and autophagy [56,66]. TnI elevation has been found to be independently correlated with LV wall thickness due to increased LV mass [67]. The increased wall stress caused by pressure and volume overload also predisposes the ventricular wall to hypertrophy and fibrosis. A thickened myocardium is thus susceptible to a myocardial demand–supply mismatch, impairing subendocardial microvascular perfusion and worsening ischemia [67,68,69]. Notably, Tn levels tend to be higher in patients with ischemic cardiomyopathies compared to those with dilated cardiomyopathies [70].

#### 5.2.3. Prognostic Implications

Tn elevations are associated with impaired hemodynamic profiles in patients with HF, including lower ejection fractions, lower cardiac indices, higher systolic pulmonary artery pressures, higher wedge pressures, higher B-Type Natriuretic Peptide (BNP) levels, and a higher clinical grading of HF [56]. In light of these multifactorial insults, a meta-analysis showed that elevated hs-TnT in chronic HF is independently associated with all-cause mortality, cardiovascular mortality, and cardiovascular hospitalization [60], and other studies showed that both conventional TnT and hs-TnT are associated with mortality and combined adverse cardiovascular outcomes [71,72].

### 5.3. Myocarditis and Pericarditis

#### 5.3.1. Epidemiology

Historical studies estimate that one-third of patients with myocarditis have abnormal values of conventional Tn. In a study including 53 patients with a histological diagnosis of myocarditis, conventional TnI was elevated in 34% of patients with myocarditis [73]. In another study of 80 patients with clinically suspected myocarditis, TnT was detected in 28 (35%) of serum samples [74]. However, these studies are several decades old. The prevalence of elevated Tn in milder cases of myocarditis is likely higher in the present day with the widespread use of hs-Tn. In a study of the transition from conventional to hs-Tn at a single center, the transition from using conventional to hs-Tn was associated with a two-fold increase in the diagnosis rate [75].

#### 5.3.2. Hypothesized Mechanism

Elevated Tn can be found in patients with myocarditis in whom CAD has been excluded [76,77]. It has been postulated that this is due, at least in part, to transient ischemia caused by coronary vasospasm [78]. Given that Tn elevation can be seen in the absence of LV dysfunction in cases of myocarditis, it is thought that Tn release is more likely due to transiently increased membrane permeability of myocyte membranes rather than true cell necrosis [79]. It has been postulated that this increased permeability and subsequent Tn release is the result of the direct cytotoxic effects of infectious agents (viruses, bacteria, etc.), exogenous toxins, and autoantibodies directed against cardiomyocytes [80].

Tn elevation can also be seen in cases of pericarditis. The prevalence of Tn elevation in pericarditis has been described as 32% [76], 49% [77], and 71% [81] in different studies prior to the introduction of hs-Tn. Notably, although Tn elevation is reported in cases of pericarditis, Tn is not found in the pericardium itself [69,81]. Therefore, Tn elevation in pericarditis likely represents the concurrent involvement of the myocardium (e.g., inflammatory involvement of subepicardial myocytes), and the presentation is better characterized as myopericarditis or perimyocarditis, with similar mechanisms of Tn release to those in myocarditis.

#### 5.3.3. Prognostic Implications

Patients with myocarditis and Tn elevation typically have similar features and outcomes to those of their counterparts without Tn elevation. In a study of 80 patients with clinically suspected myocarditis with and without elevated values in conventional TnT assays, the frequency of clinical symptoms was equal in both groups, with no differences in the frequency of AF, premature supraventricular beats, premature ventricular beats, bundle branch block, or ST-segment alterations. Hemodynamic variables at rest measured by ventriculography (ejection fraction, end-diastolic volume, end-systolic volume, stroke volume) also did not differ by Tn elevation status. The only difference was that patients with elevated TnT had more frequent pericardial effusions (*p* = 0.024) [74].

Outcomes are also similar for patients with and without Tn elevation in pericarditis and myopericarditis. In a study of 69 patients with acute pericarditis, Tn elevation was associated with increased rates of ST elevation at the time of illness; however, no differences in symptom intensity, disease severity, or the initial length of hospital stay were observed based on conventional Tn levels [77]. Furthermore, long-term outcomes are similar in patients with pericarditis with and without Tn elevation, with one study of 118 patients with acute pericarditis showing no cases of cardiac tamponade or residual LV dysfunction in either group [76]. In myopericarditis, while Tn elevation is believed to be associated with the extent of myocardial inflammatory involvement, it has not been associated with an adverse prognosis. In a study of 486 patients with acute pericarditis or myocarditis, the majority of patients with and without conventional Tn elevation had normalized findings on echocardiogram, ECG, and treadmill testing at 36 months [82].

### 5.4. Endocarditis

#### 5.4.1. Epidemiology

The prevalence of elevated Tn in the setting of endocarditis is relatively high and has been observed to be between 57% and 84% [83,84,85,86].

#### 5.4.2. Hypothesized Mechanism

Endocardial myocytes do not contain Tn. As previously discussed in cases of pericarditis, it has been hypothesized that serum Tn elevations in endocarditis occur due to inflammation of the surrounding myocardial tissue [80].

#### 5.4.3. Prognostic Implications

In a meta-analysis of nine observational studies, conventional and hs-Tn elevation in endocarditis was associated with a significantly increased risk of in-hospital mortality and the need for surgery or valve replacement [86]. Additionally, patients with Tn elevation experienced significantly higher rates of cardiac abscesses and ischemic or hemorrhagic cerebral events [86]. However, it is important to consider that the findings of this meta-analysis could have been confounded by the relationship between Tn release and greater degrees of acute illness, as the constituent studies did not systematically account for the presence of cardiogenic shock, severe HF, or other comorbidities that could affect the prognosis.

### 5.5. Hypertensive Emergency

#### 5.5.1. Epidemiology

Elevations in Tn are frequently encountered in patients with a hypertensive emergency. One study of 467 patients presenting to the emergency department with hypertensive urgency or emergency found detectable Tn using conventional assays in 35% of patients. However, myocardial injury, as defined by elevation above the 99th URL, occurred in fewer than half of this subset of patients, and even fewer patients experienced dynamic changes in troponin values suggestive of an acute process [87]. On the other hand, the prevalence of Tn elevation may be higher in the inpatient setting. A prospective study of 205 adults admitted for hypertensive emergencies at a single center found that 49.8% had elevated conventional TnI. However, those with elevated troponins also had markedly higher serum creatinine values (0.89 mg/dL vs. 2.07 mg/dL, *p* < 0.001), making decreased renal function a possible confounding factor in this study [88].

#### 5.5.2. Hypothesized Mechanism

The mechanisms of Tn release in a hypertensive emergency are incompletely understood and often confounded by comorbidities such as HF or chronic kidney disease [87,88]. The precise mechanism of Tn release likely varies due to the diversity of pathophysiologic conditions that fulfill the criteria for a diagnosis of a hypertensive emergency. For instance, in patients experiencing hypertension-related cardiac manifestations such as an ACS or type 2 MI, elevations in serum Tn may be the direct result of endomyocardial necrosis [89]. In the setting of HF, a common end-organ manifestation of a hypertensive emergency, increased intraventricular pressures and resultant rises in wall stress are known to lead to the release of Tn, even in the absence of coronary disease [57,62]. Hypertension-induced endothelial dysfunction may play a role, as it has been hypothesized to cause Tn release through various proinflammatory, thrombotic, and ischemic downstream effects [90,91]. Although our understanding of the cause of endothelial dysfunction during episodes of severe hypotension is limited, it may be partially mediated by increased renin–angiotensin–aldosterone (RAAS) system activity [89,92].

#### 5.5.3. Prognostic Implications

In multiple studies, elevations of both conventional and hs-TnI have been found to be associated with worse outcomes, including higher rates of pulmonary edema, intubation, and mortality [88,93,94]. Patients with higher hs-TnI levels were observed to have higher initial presenting SBP, more abnormal laboratory findings (including creatinine, BNP, D-dimer, and hemoglobin), and higher rates of admission, revisit, and readmission [93]. In a prospective study of 918 consecutive patients who presented to the emergency department with a hypertensive emergency and without ACS, elevated hs-TnI was found to have a strong association with mortality independent of age, sex, comorbidity burden, and clinical markers of adverse physiology [94]. In a retrospective analysis of 171 patients presenting to the ED with a hypertensive emergency or urgency, higher conventional TnI levels were also found to be associated with a substantially increased risk of major adverse cardiac and cerebrovascular events (hazard ratio = 2.77, *p* < 0.001) [95].

### 5.6. Acute Aortic Syndromes

#### 5.6.1. Epidemiology

In a meta-analysis of four studies and 496 patients with acute aortic dissection (AAD), predominantly type A, elevated Tn measured by conventional assays was present in 26.8% of patients with AAD and ranged between 23% and 33% in individual studies [96]. Meanwhile, in a study that made use of hs-Tn assays, elevated hs-TnT was seen in 61.2% of patients who presented with type A aortic dissection [97].

#### 5.6.2. Hypothesized Mechanism

The aortic wall contains the calcium-binding protein calponin but not Tn [98,99], suggesting that the mechanism of Tn release in AAD is likely multifactorial and may include coronary artery obstruction, acute LV pressure overload, and shock. A study of 398 patients with AAD found that the presence of Tn elevation (both conventional and hs-Tn) during AAD was frequently associated with ACS-like ECG abnormalities, with 13% of patients with abnormal Tn presenting with classic ST elevation [100]. Among 10 patients who underwent transesophageal echocardiograms, 4 had an anatomic obstruction of at least one coronary artery due to coronary dissection or diastolic apposition of the flap to the ostium, illustrating at least one mechanism of myocardial ischemia and likely consequent Tn release [100].

#### 5.6.3. Prognostic Implications

The association of elevated Tn with mortality in aortic dissection is unclear. In the aforementioned study, the combination of Tn elevation and ACS-like ECG findings was associated with a two-fold increased risk of in-hospital diagnostic delay and a significantly increased risk of the composite endpoint of coronary angiography, antithrombotic therapy, or in-hospital diagnostic delay. Nevertheless, the in-hospital diagnostic delay did not influence mortality [100]. In contrast, a meta-analysis of five studies and 711 patients and a study of 103 patients with type-A AAD showed an association between Tn elevation (conventional or hs-Tn) and short-term mortality [96,97].

### 5.7. Stress (Takotsubo) Cardiomyopathy

#### 5.7.1. Epidemiology

Most patients with stress (takotsubo) cardiomyopathy have a modest rise in Tn [101,102]. In a study of 59 patients with stress cardiomyopathy, 95% of patients had elevations in conventional TnT [103]. Patients with stress cardiomyopathy can have chest pain similar to ischemic chest pain and ST-segment and T-wave changes on ECG, which can make the condition difficult to differentiate from ACS. However, the magnitude of the increase in serum Tn is not as pronounced as that observed in ACS. For example, in a study of 136 patients with stress cardiomyopathy using conventional Tn, median TnT was 3.88 ng/mL in patients with left anterior descending artery occlusions and 0.64 ng/mL in patients with stress cardiomyopathy [103]. In another study of 41 patients with ACS and 51 patients with suspected stress cardiomyopathy, median hs-TnT was 564.3 pg/mL in patients with ACS and 162.0 pg/mL in patients with stress cardiomyopathy [104].

#### 5.7.2. Hypothesized Mechanism

The pathophysiology of Tn release in stress cardiomyopathy is not well understood. Proposed mechanisms include catecholamine-induced myocardial stunning, coronary vasospasm-induced ischemia, and focal myocarditis [105]. It is well known that stress cardiomyopathy is mediated by supraphysiologic levels of plasma catecholamines and stress-related neuropeptides [106]. The apical myocardium may have increased responsiveness to sympathetic stimulation, while there is a relative sparing of the basal segments. An alternative hypothesis is ischemia-mediated stunning due to coronary vasospasm. Microvascular dysfunction is present in at least two-thirds of patients at the time of presentation, and its severity correlates with the magnitude of Tn elevation and ECG abnormalities [107]. Others have noted that the degree of Tn elevation in stress cardiomyopathy is disproportionately low compared to the large territory of dysfunctional myocardium on echocardiography, suggesting that mechanisms other than myocyte necrosis are involved in Tn release [102]. This hypothesis is supported by the absence of late gadolinium enhancement on cardiac MRI both during the acute phase and on follow-up imaging, in contradistinction to ischemia or myocarditis [102].

#### 5.7.3. Prognostic Implications

Studies have shown that Tn elevation in stress cardiomyopathy has an independent association with long-term adverse outcomes, including increased mortality [108,109]. Additionally, in a study of 1750 patients with stress cardiomyopathy, elevations in a combination of conventional TnI, conventional TnT, and hs-TnT more than 10 times the 99% percentile URL were significantly associated with an increase in the composite endpoint of in-hospital complications, which included catecholamine use, cardiogenic shock, invasive or noninvasive ventilation, cardiopulmonary resuscitation, and death [110].

### 5.8. Amyloidosis

#### 5.8.1. Epidemiology

Persistently elevated Tn levels are frequently found in amyloidosis, including both primary/light chain (AL) and transthyretin (TTR) amyloidosis. In a study of 117 patients with cardiac amyloidosis, 64.1% had detectable conventional TnI, defined as ≥0.06 ng/mL [111]. In another study of 102 patients with cardiac amyloidosis, 88.23% had an elevated hs-TnT of >14 ng/L, with the lower limit of detection being 1 ng/L [112]. In another study comparing 96 patients with cardiac amyloidosis (AL, wild-type TTR, and mutant TTR amyloidosis) and 91 patients with non-amyloid causes of cardiac hypertrophy, hs-TnT levels were significantly higher in the cardiac amyloidosis group than in the other hypertrophy group [113].

The degree of elevation may also relate to organ involvement. In a study of 163 patients with AL amyloidosis, hs-TnT was highest in patients with apparent cardiac involvement, followed by patients with suspected cardiac involvement, followed by patients with no apparent cardiac involvement. However, even in AL amyloidosis patients with no apparent cardiac involvement, median hs-TnT levels were above the 99th percentile, underscoring the high prevalence of elevated Tn in amyloidosis [114].

#### 5.8.2. Hypothesized Mechanism

The mechanism of Tn release in patients with cardiac amyloidosis is thought to be multifactorial. Proposed mechanisms include microvascular ischemia due to luminal stenosis and extrinsic compression of microvasculature in the setting of amyloid deposits [115,116]. In a study of 96 patients with cardiac amyloidosis, 66% had obstructive coronary amyloidosis, and microscopic changes of myocardial ischemia were more common in patients with intramural coronary amyloidosis [116]. Additionally, amyloid protein and its precursor can have direct proinflammatory or toxic effects that can lead to myocardial cell damage, membrane leakage, and Tn release [117,118]. Finally, increased LV filling pressure and wall stress due to diastolic dysfunction in amyloidosis likely also contribute to Tn release, which has been demonstrated in multiple studies [56,63,64].

#### 5.8.3. Prognostic Implications

The degree of Tn elevation in amyloidosis may be associated with higher mortality. Using conventional assays, a study of 98 patients with AL amyloidosis undergoing peripheral blood stem cell transplantation found that elevations in TnI, but not TnT, were associated with poorer survival in the 90 days post-transplant [119]. Using hs-Tn assays, a study of 163 patients with AL amyloidosis found that hs-TnT > 50 ng/L was associated with poorer survival but that the survival of patients with hs-TnT of 14–50 ng/L did not differ from that of patients with hs-TnT of 3–14 ng/L. The association between hs-TnT and mortality persisted after the exclusion of patients with impaired renal function [114].

### 5.9. Heart Transplant

#### 5.9.1. Epidemiology

Tn elevation occurs in virtually all patients in the post-heart transplant period. In one study of 110 patients who received a heart transplant, all patients had elevated conventional TnI levels during the first month after transplant, and in 51% of patients, TnI remained persistently elevated after 12 months [120]. More recently, in a study of 170 cardiac transplant recipients who underwent hs-TnI measurement serially 10–12 times within the first year after transplant, detectable hs-TnI levels were found in all samples, and 82% of the samples had hs-TnI levels above the normal range [121].

#### 5.9.2. Hypothesized Mechanism

In the immediate post-transplant period, myocytes are commonly subject to ischemic injury and reperfusion injury, leading to coagulative myocyte necrosis [120]. However, many patients have persistent cardiac Tn elevation lasting over a month. However, because the half-life of TnT is 2 h, the persistence of Tn elevation suggests the existence of processes other than perioperative ischemic damage, such as host immunity against the transplanted heart, that continue to injure myocytes [122].

#### 5.9.3. Prognostic Implications

Post-transplant Tn levels have been shown to be associated with short-term and long-term mortality. In a study of 212 heart transplant recipients, elevations in hs-TnT measured 48 h postoperatively were associated with increased all-cause mortality at 1 year [123]. In another study of 156 heart transplant recipients, elevations in hs-TnI measured a median of 10 months post-transplant were associated with increased long-term all-cause mortality at a median follow-up of 10 years [124].

The role of post-transplant Tn levels in assessing the presence of cardiac transplant rejection is less clear. In a recent meta-analysis of 27 studies with 1684 heart transplant recipients, patients with acute rejection had a statistically significant late elevation in Tn measurements taken at least 1 month postoperatively (the analysis included both conventional and hs-Tn assays and both TnI and TnT assays) [125]. However, the pooled diagnostic accuracy was poor, with a sensitivity of 41% and specificity of 76%, suggesting that Tn is insufficient for use as a stand-alone diagnostic tool. In a study of 110 heart transplant recipients, the presence of persistent conventional TnI elevation measured at 12 months was associated with the presence of fibrin deposits in the microvasculature and cardiomyocytes [120]. Patients with persistently elevated levels of conventional TnI had an increased risk for the development of CAD and graft failure [121]. In the aforementioned recent study of 170 patients who underwent routine surveillance endomyocardial biopsy, there was no association between hs-TnI and the presence of acute cellular rejection on endomyocardial biopsy [121]. Taken together, the current literature suggests that Tn elevation has limited reliability as a criterion for assessing transplant rejection.

### 5.10. Cardiac Procedures (e.g., Ablation, Cardioversion)

#### 5.10.1. Epidemiology

Unsurprisingly, Tn elevation has been seen in the majority of patients undergoing cardiac ablation. In a study of 60 patients undergoing radiofrequency (RF) ablation by pulmonary vein isolation for AF who had no underlying structural heart disease and baseline normal conventional TnT, all patients were found to have increased post-procedure Tn, with all measurements exceeding the diagnostic threshold for MI [126]. In a study of 51 patients undergoing RF ablation for different indications using conventional TnI assays, the lowest release of TnI was found in ablation for atrioventricular nodal reentrant tachycardia, and the highest release of TnI was found in ablation for AF or AFL [127]. In another study of patients undergoing ablation for ventricular tachycardia (VT) (19 patients) and AF (24 patients), the release of hs-TnT was seen in both groups but reached higher values in VT, though levels equalized after 24 h [128].

Surprisingly, Tn elevation in elective external cardioversion is less common. Many older studies found no significant increase in conventional Tn levels after cardioversion, and in the few patients who had a Tn increase post-cardioversion, the increase was usually mild [129,130,131,132,133]. More recently, a study of hs-TnT in 120 patients who underwent elective external cardioversion for AF or AFL [134] and a study of hs-TnI in 171 patients who underwent elective external cardioversion for AF [135] found that although Tn was detectable in most patients using an hs-Tn assay, it was within the normal range (under 99th percentile), and there was no significant difference between pre- and post-cardioversion hs-TnT.

#### 5.10.2. Hypothesized Mechanism

It is hypothesized that RF catheter ablation creates a small area of localized necrosis, causing Tn release through direct myocardial damage from the procedure itself [127]. Since external cardioversion is not associated with Tn elevation, no mechanism is provided.

#### 5.10.3. Prognostic Implications

In a study of patients who underwent AF ablation, the degree of conventional TnT elevation was not related to the number of RF lesions, RF time, procedure time, or associated external cardioversion [126]. Interestingly, the degree of Tn elevation after RF catheter ablation is associated with favorable outcomes, greater reversal of structural remodeling, a lower likelihood of the need for repeat RF ablation, and an increased reduction in the left atrial volume index at 6 months. These findings may be due to elevated TnT being reflective of the successful ablation of the offending arrhythmia-inducing cardiomyocytes [136]. On the other hand, the less common, mild elevation in Tn after cardioversion has not been shown to have any prognostic significance, with no association between hs-TnI levels and AF recurrence after cardioversion [135].

### 5.11. Pulmonary Embolism

#### 5.11.1. Epidemiology

Pulmonary embolism (PE) has been reported to be the most common non-cardiac cause of increased Tn [137], with an estimated 10–50% of patients with PE presenting with elevated conventional Tn [138,139]. Studies investigating the release kinetics of conventional TnT have shown that peak TnT in PE tends to be lower and persists for a shorter time compared to ACS [140]. Using hs-Tn assays, one study of 834 patients with hemodynamically stable PE found the prevalence of hs-TnI to be 31.7% [141], and another study of 4611 patients with PE found the prevalence of hs-TnT to be 76.5% (though this study did not exclude patients with preexisting cardiac conditions) [142].

#### 5.11.2. Hypothesized Mechanism

The mechanism of Tn elevation in PE is thought to be related to acute right ventricular (RV) strain and myocardial ischemia secondary to an increase in pulmonary artery resistance [143,144]. This elevation is typically modest, but it typically reflects the amount of myocardium injured by ischemia [138].

#### 5.11.3. Prognostic Implications

Conventional Tn elevations in PE are associated with an increased risk of a complicated in-hospital course, including prolonged hypotension, cardiogenic shock, the need for resuscitation, and death [139,140,141,142,145,146,147,148]. In a meta-analysis of 20 studies of acute PE, patients with elevated conventional TnT or TnI had a 5-fold increase in mortality compared to patients without elevated Tn (19.7% vs. 3.7%) [147]. Additionally, RV dysfunction increases the risk for adverse clinical outcomes in all patients, but this risk is 10-fold higher in the presence of elevated conventional TnT (>0.4 ng/mL). Some have suggested that this increased risk may warrant more aggressive treatment approaches, such as thrombolysis or embolectomy, in patients with PE and elevated TnT [149]. Using hs-Tn assays, hsTnT has been found to be associated with both short-term and long-term mortality [142], though studies show mixed results on the prognostic effects of elevated hsTnI [141,150].

### 5.12. Pulmonary Hypertension

#### 5.12.1. Epidemiology

Tn elevations can be found in patients with pulmonary hypertension (PH). In a cohort study of 55 patients with mixed classes of PH, elevated Tn was seen in approximately 27% and 90% of patients using hs-TnT and hs-TnI assays, respectively, and in 27% and 11% of patients using fourth-generation TnT and TnI assays, respectively [151].

#### 5.12.2. Hypothesized Mechanism

Tn elevation in PH is likely secondary to RV injury. Elevated pulmonary vascular resistance leads to increased RV tension, which may cause RV injury and ischemia [152]. In fact, right chamber dilation was more common in patients with PH and detectable conventional Tn levels. Furthermore, correlations between conventional TnI and C-reactive protein (CRP) have been reported, suggesting a possible inflammatory component in TnI elevation [152].

#### 5.12.3. Prognostic Implications

Elevated Tn in PH is associated with adverse outcomes. Specifically, in a cohort study of 68 patients with group 1 PH, patients with detectable conventional TnI had worse functional class, lower 6 min walking distance, more evidence of right heart strain on an echocardiogram, higher levels of BNP, and worse lung-transplant-free survival compared to patients with undetectable TnI [153]. Similar findings were seen in studies evaluating conventional TnT and hs-TnT in patients with mixed classes of PH. Specifically, patients with detectable conventional TnT or hs-TnT had higher heart rates, higher BNP levels, shorter 6 min walk tests, more right heart strain, and worse 2-year cumulative survival (29% vs. 81%) [151,152]. In a meta-analysis of eight studies with 739 patients, elevated conventional Tn in general conferred a higher mortality risk, with TnI predicting mortality better than TnT [154].

## 6. Non-Cardiovascular Etiologies of Troponin Elevation

**Figure 4:**
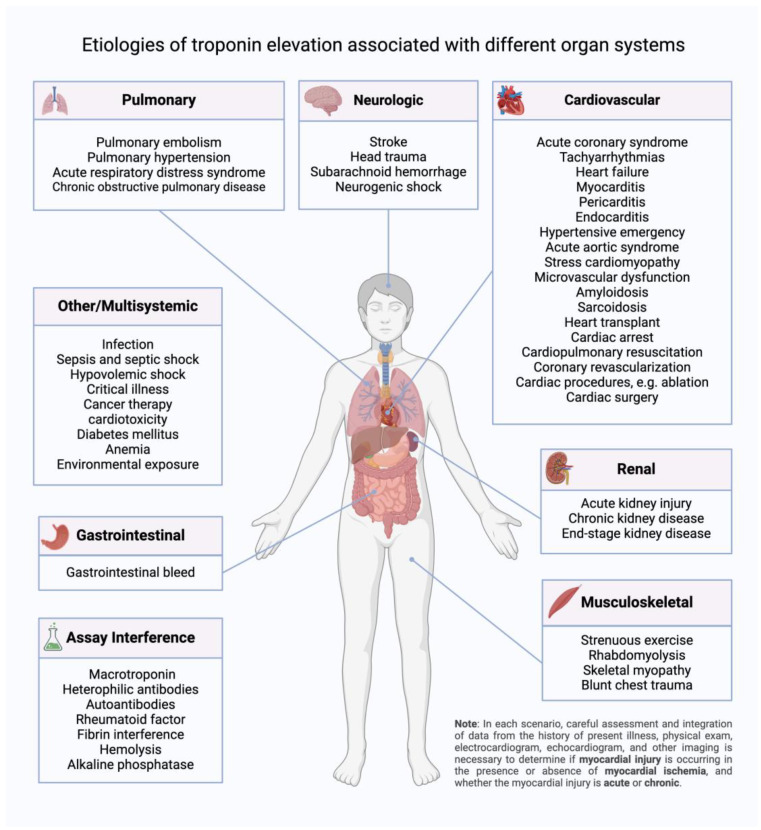
Etiologies of troponin elevation associated with different organ systems. Original figure designed in BioRender.

Although Tn is commonly elevated in ACS and cardiac disorders, it can also be elevated due to non-cardiac etiologies, including pulmonary, renal, neurologic, musculoskeletal, oncologic, and gastrointestinal causes, as well as acute illness and trauma (Figure 4). The pathophysiology of elevated Tn due to non-cardiac causes is not completely clear in some cases, but elevated Tn correlates closely with a poor prognosis in most of these conditions, which should be considered in the differential diagnosis of patients with elevated Tn and no clear cardiac cause [155]. The possibility of ACS should be considered alongside non-ACS causes of myocardial injury in each of the conditions below, using clinical judgment based on findings from the history, physical exam, ECG, and imaging data, such as an echocardiogram.

### 6.1. Acute Respiratory Distress Syndrome

#### 6.1.1. Epidemiology

The prevalence of Tn elevation is high in acute respiratory distress syndrome (ARDS). In a study of the Fluids and Catheters Treatment Trial (FACTT) and the Assessment of Low tidal Volume and elevated End-expiratory volume to Obviate Lung Injury (ALVEOLI) trial, which together enrolled 1057 patients with ARDS without signs or symptoms of acute cardiac ischemia, 94% of ARDS patients had detectable hs-TnI levels, and 56% had hs-TnI levels above the 99th percentile of a healthy reference population [156].

#### 6.1.2. Hypothesized Mechanism

In the aforementioned study, the mechanism of Tn elevation in ARDS was surmised to include myocyte necrosis in the setting of critical illness and/or cellular changes in myocytes without necrosis, including increased myocyte permeability, cell membrane changes, and the cellular release of Tn degradation products [156]. It should be noted that in both the FACTT and ALVEOLI trials, patients were excluded from enrollment if they had signs or symptoms of acute cardiac ischemia. This suggests that the Tn elevation in these patients with ARDS occurred via myocardial injury rather than myocardial infarction.

#### 6.1.3. Prognostic Implications

Elevated hs-Tn may be associated with increased morbidity and mortality in ARDS patients [157]. In the study of the FACTT and ALVEOLI trials, increased hs-Tn levels in ARDS were also associated with markers of other organ dysfunction, such as elevated serum creatinine, the Sequential Organ Failure Assessment (SOFA) score, ventilation indices like pH and pCO_2_, vital signs such as heart rate and body temperature, and abnormal findings on echocardiography including tricuspid regurgitation and regional wall motion abnormalities. However, after adjusting for clinical factors like the SOFA score, heart rate, and vasopressor use, hs-Tn was no longer independently associated with mortality, suggesting that the degree of Tn elevation may simply be a reflection of the underlying stressors of critical illness rather than an active player in the pathogenesis of deterioration [156].

### 6.2. Chronic Obstructive Pulmonary Disease

#### 6.2.1. Epidemiology

Tn elevation is common in chronic obstructive pulmonary disease (COPD). Patients with COPD tend to have modestly higher hs-Tn levels at baseline (hs-TnT of 7.75 ng/L) compared to patients without COPD (3.01 ng/L). Additionally, hs-TnT levels tend to be higher in acute COPD exacerbations compared to stable disease, and hs-TnT levels are higher with increasing classes of COPD [158].

#### 6.2.2. Hypothesized Mechanism

Elevated Tn levels in COPD may be a result of hypoxemic pulmonary vasoconstriction, which leads to elevated pulmonary pressure and, consequently, increased RV stretch, strain, and possible myocardial necrosis. This dysfunction is similar to the pathophysiology that occurs secondary to PE [159,160].

#### 6.2.3. Prognostic Implications

Elevated hs-Tn is associated with increased mortality in patients with COPD. In a study of 2741 patients with COPD, elevated hs-TnI levels were an independent prognostic factor for mortality after discharge, regardless of the data analysis methodology and general cardiovascular risk [160]. A cohort study of 1599 patients with COPD further demonstrated a positive association between hs-TnI levels and the risk of cardiovascular events and death [161]. Furthermore, a cohort study of 99 patients hospitalized for COPD exacerbation showed that this association was modified by the heart rate at admission, with a stronger association demonstrated between mortality and hs-TnT in patients with tachycardia [162].

### 6.3. Chronic Kidney Disease and End-Stage Kidney Disease

#### 6.3.1. Epidemiology

Persistently elevated Tn is common in patients with end-stage kidney disease (ESKD), even when there is no suspected myocardial ischemia. The prevalence of Tn elevations in ESKD depends on the Tn assay used. For example, in patients with ESKD and no clinical or reported evidence of cardiovascular disease, conventional TnT elevations can be found in up to 53% of patients, and hs-TnT may be detectable in up to 81–99% of patients, while conventional TnI and hs-TnI elevations are less common, found is as few as 6% and 34% of patients, respectively [163,164,165,166].

#### 6.3.2. Hypothesized Mechanism

Both increased cardiac release and decreased clearance have been implicated as possible mechanisms for increased Tn in ESKD. A study examining the clearance of conventional TnT in rats and humans found that at high concentrations of TnT (e.g., as occurs after a large MI), the extrarenal clearance of TnT dominates, while at low concentrations of TnT (e.g., in patients with chronic kidney disease [CKD]), renal clearance also contributes to TnT clearance [167]. In addition, increased cardiac release of Tn may occur due to cardiac abnormalities resulting from ESKD, such as increased ventricular pressures, small-vessel coronary obstruction, anemia, hypotension, and direct toxic effects on the myocardium from uremia [1].

#### 6.3.3. Prognostic Implications

Because increased Tn is likely reflective of myocardial injury, it can be an important prognostic indicator. Several early studies, including a meta-analysis of 28 studies and a retrospective study of 733 ESKD patients, showed that elevated conventional TnT (≥0.1 μg/L) is associated with increased cardiovascular and all-cause mortality [168,169]. Similarly, several studies have shown that elevated hs-TnT and hs-TnI are associated with increased risk for long-term major adverse cardiovascular events (MACE), and hs-TnT is associated with increased mortality [170,171,172]. 

Diagnosing acute MI in patients with CKD and ESKD can be challenging due to the persistently elevated nature of Tn in these patients. Studies have found that static cutoffs for hs-TnI and hs-TnT have low specificity in detecting NSTEMI in CKD and ESKD patients, in whom Tn can be chronically elevated even without acute myocardial injury. Instead, serial Tn measurements can be used in these patients for higher diagnostic accuracy of acute MI, and changes in ECG or imaging and clinical judgment must be considered for diagnosis. It is important to remember that a lack of increase in serial Tn levels may make acute MI less likely but does not indicate the absence of baseline CAD, as renal dysfunction and CAD are correlated [5,173].

### 6.4. Acute Kidney Injury

#### 6.4.1. Epidemiology

Elevated Tn may be seen in patients with acute kidney injury (AKI) alone, without other conditions known to cause elevated Tn. In a cohort study of 19 patients with AKI (excluding patients with concomitant multi-organ failure, acute MI, myocarditis, pericarditis, infiltrative cardiac disease, arrhythmia, PE, congestive HF, and sepsis), elevated conventional TnT and TnI were found in 30% of patients. These elevations were more common in certain scenarios, including older age, a history of ischemic heart disease, and abnormal ECG [174].

#### 6.4.2. Hypothesized Mechanism

Tn elevation in patients with AKI but no cardiac disease may be due to the decreased renal clearance of normally released Tn [174,175]. In patients with comorbid conditions, it is likely that factors that precipitate AKI also precipitate myocardial injury, leading to Tn release. Of note, kidney function decreases during AKI and increases during the recovery phase, leading to a rise-and-fall pattern in Tn that may mimic acute MI [176].

#### 6.4.3. Prognostic Implications

The significance of elevated Tn in AKI without confounding comorbidities is not well studied.

### 6.5. Stroke

#### 6.5.1. Epidemiology

Tn elevations may be seen in all types of stroke, including ischemic stroke and intracerebral or subarachnoid hemorrhage. Tn elevations are found in 30–60% of patients with acute ischemic stroke (hs-Tn) [177,178,179,180], 11–52% of patients with subarachnoid hemorrhage (conventional Tn) [181,182], and 41% of intracerebral hemorrhages (conventional TnI) [183]. Several comorbidities increase the risk of hs-TnT elevation after stroke, including older age, structural or coronary heart disease, and impaired renal function [179,184,185].

#### 6.5.2. Hypothesized Mechanism

It is thought that Tn elevations in stroke are a result of the activation of the sympathoadrenal system and increased catecholamine release, which can lead to direct myocardial toxicity, demand ischemia, LV dysfunction, arrhythmias, possible plaque instability causing MI, and neurogenic sudden cardiac death [177,186,187,188].

#### 6.5.3. Prognostic Implications

Elevations in Tn increase the risk of adverse outcomes after stroke. In ischemic stroke, hsTnT and TnI elevations are associated with s decline in cognitive function and an increased risk of cardiac complications, including reduced LV function, arrhythmias, and MACE [178,186,189,190]. In subarachnoid hemorrhage, several meta-analyses showed that conventional Tn elevations were associated with increased mortality, more delayed cerebral ischemia, and worse neurologic status [181,182,187]. Adverse outcomes of Tn elevations in intracerebral hemorrhage are not as well studied but may be associated with worse functional status and increased mortality [191,192].

### 6.6. Sepsis and Septic Shock

#### 6.6.1. Epidemiology

Tn elevations are common in critically ill patients, including patients with sepsis and septic shock. It is estimated that 31–80% of patients with systemic inflammatory response syndrome (SIRS; the study was conducted prior to the phasing out of the SIRS definition), sepsis, or septic shock have elevated conventional TnT and TnI levels [193]. Using hs-Tn assays, one study found elevated hs-TnI in 60% of patients (excluding patients with other apparent causes of Tn elevation) [194], and another found hs-TnI elevations in 47% of patients (excluding post-cardiac arrest patients) [195].

#### 6.6.2. Hypothesized Mechanism

The mechanism of Tn elevation in sepsis and septic shock in the absence of ACS is not fully understood but may be related to a myocardial oxygen demand–supply mismatch, microvascular dysfunction, increased myocardial cell membrane permeability, and the presence of myocardial depressive factors like inflammatory mediators [193].

#### 6.6.3. Prognostic Implications

Tn elevations in sepsis and septic shock are associated with an increased risk of cardiovascular complications, even in patients without preexisting cardiovascular disease. In a retrospective study of over 14,000 patients with sepsis and no CAD, elevated conventional TnI was found to be associated with an increased risk for the development of atherosclerotic cardiovascular disease, atrial fibrillation, and HF [196]. Furthermore, several studies have found associations between elevated Tn (conventional Tn and hs-TnI) in sepsis and mortality [194,197,198].

### 6.7. Extreme Exercise

#### 6.7.1. Epidemiology

Exercise-induced Tn elevation is a known phenomenon. For example, in a systematic review of 16 studies (936 participants), 0.6% of participants had detectable conventional Tn prior to running a marathon, while 62% of post-marathon participants were found to have detectable Tn, and 15% were found to have Tn above the myocardial necrosis threshold [199]. Two recent studies using hs-TnT and hs-TnI found that all marathon runners had detectable levels after a race [200,201].

#### 6.7.2. Hypothesized Mechanism

A proposed etiology for the Tn increase in exercise is increased myocyte cell membrane permeability, likely due to transient wounding of the sarcolemma or stress-mediated integrin-stimulated Tn release [202].

#### 6.7.3. Prognostic Implications

The clinical relevance of Tn elevation after exercise is not clear [203]. Many studies have found no association between elevated post-exercise Tn (in both healthy patients and patients with angina) and an increased risk of adverse outcomes [204,205,206,207]. One recent study found an association between detectable conventional Tn after long-distance walking and an increased risk of long-term MACE, although the sample consisted of middle-aged adults, making the findings inapplicable to younger athletes [208]. Because of the unclear significance of elevated Tn after exercise, it is suggested that patients seeking care after exercise who are found to have elevated Tn be evaluated for ACS using the standard protocol [202].

### 6.8. Blunt Chest Trauma

#### 6.8.1. Epidemiology

The reported incidence of cardiac injury following blunt chest trauma is variable, largely due to a lack of standardized diagnostic criteria. Therefore, Tn elevations are also variable based on the classification of the trauma. Using conventional Tn assays, various studies have found detectable levels of TnI in 44–50% and elevated levels of TnI and/or TnT in 23–43% of patients with blunt chest trauma [209,210]. Using high-sensitivity assays, a study of 82 patients with blunt chest trauma found detectable levels of hs-TnT in 34% and elevated levels in 66% of patients [211].

#### 6.8.2. Hypothesized Mechanism

Six potential mechanisms have been suggested for blunt cardiac injury: direct (the most common), indirect, bidirectional, deceleration, blast, crush, concussive, or combined [212]. The most common cardiac injuries from blunt trauma resulting in death are due to transmural cardiac chamber rupture, venous–atrial confluence tears, or coronary artery tears or dissection [213].

Direct trauma and damage to cardiac structures can result in the loss of integrity of cardiac myocytes, resulting in Tn release. Tn release may also happen more indirectly through increases in intracardiac pressure. Indirect increases in cardiac preload from the abdominal or extremity veins cause a sharp increase in intracardiac pressure and subsequent myocardial injury. This type of injury is most likely at the end of diastole, when the ventricles are already maximally dilated [214]. This suggests that an increase in intracardiac pressure through these multiple mechanisms is another etiology of Tn release, which is consistent with multiple studies showing that Tn release is associated with increased wall stress and myocyte stretch [63,64,65].

Furthermore, sequelae of trauma, including shock, hypoxia, thermal injury, and sepsis, can contribute to Tn release [215]. In fact, in a large series of 1081 patients, the degree of TnI elevation was more strongly related to the degree of overall injury and physiological stress than to mechanical chest trauma itself [216].

#### 6.8.3. Prognostic Implications

Higher Tn levels are associated with significantly higher injury severity scores, the need for pressors, and mortality [216,217]. Multiple studies have shown that in patients with normal ECG findings and serial measurements of conventional TnI within the reference interval, there was no development of significant blunt thoracic trauma (defined as cardiogenic shock, arrhythmias requiring treatment, structural cardiac abnormalities directly related to the cardiac trauma) [209,210]. In one study of 115 patients with blunt chest trauma, ECG and conventional TnI had positive predictive values of 28% and 48% and negative predictive values of 95% and 93%, respectively, for significant blunt cardiac injury. However, when both tests were concordant (both abnormal or both normal), the positive and negative predictive values increased to 62% and 100%, respectively [209]. Using hs-Tn assays, elevated hs-TnT is associated with higher in-hospital mortality, a higher number of ventilator days, and lower Glasgow Outcome Scale scores on discharge [211]. Therefore, in the absence of other injuries or hemodynamic instability, patients with normal ECG and Tn can be discharged, whereas increased Tn may serve to identify patients at increased risk of mortality.

### 6.9. Rhabdomyolysis

#### 6.9.1. Epidemiology

Patients presenting with rhabdomyolysis may have elevated conventional TnI levels in 11–30% of cases [218,219,220]. In one study using hs-Tn assays, hsTnT and hsTnI levels were elevated in 63.5% and 41.6% of patients with rhabdomyolysis, respectively (although this study did not exclude patients with preexisting cardiac disease) [221].

#### 6.9.2. Hypothesized Mechanism

In patients without ACS, the etiology of Tn elevation in rhabdomyolysis is unclear. Proposed mechanisms include direct injury from free radicals, acidemia, or cytokines; hypotension; and myocardial stretch from aggressive fluid resuscitation [219]. Others propose that these are false-positive elevations that may represent minor cardiac injury or cross-reactivity with skeletal forms of Tn [218].

#### 6.9.3. Prognostic Implications

Studies on the risks of elevated conventional Tn in rhabdomyolysis show mixed results regarding the increased risk of mortality, although a large study of 404,369 patients with rhabdomyolysis showed an increased risk of mortality and a higher hospital cost in patients with elevated Tn [219,222]. Treatment with fluid resuscitation has been found to reduce measured serum Tn levels to baseline, but patients at risk for CAD should undergo evaluation after the resolution of rhabdomyolysis [155,221].

### 6.10. Skeletal Myopathy

#### 6.10.1. Epidemiology

Many patients with hereditary or acquired skeletal myopathy have elevated Tn on testing. In two studies of patients with various types of skeletal myopathy and no known cardiac disease, elevations in hs-TnT were common (68% and 69%) [223,224]. On the other hand, the percentage of hs-TnI elevation is less common and tends to be similar to that of the general population [223,225].

#### 6.10.2. Hypothesized Mechanism

Discussion is still ongoing regarding the etiology of elevated Tn in myopathy. Some suggest that cardiac Tn is re-expressed in skeletal muscles after injury and is released into the bloodstream, while others posit that skeletal TnT can cross-react with cardiac TnT and lead to false-positive Tn elevation [223,226]. In skeletal myopathies without cardiac involvement, the source of elevated TnT is skeletal rather than cardiac and is therefore less likely to be indicative of cardiac involvement in the absence of a change in serial Tn measurements [223,227]. On the other hand, isoforms of skeletal and cardiac TnI are unique, and TnI is rarely elevated in patients with skeletal myopathy and no known cardiac disease. Therefore, TnI elevations are typically seen in myopathies that tend to have cardiac involvement and can reflect myocardial fibrosis, myocardial inflammation, and recurrent, focal microvascular ischemia [223,228].

#### 6.10.3. Prognostic Implications

The prognostic implications of elevated Tn in skeletal myopathy are not well studied. One study of 142 patients with idiopathic inflammatory myopathy (IIM) found that elevated levels of hs-TnI in IIM patients were associated with cardiac involvement. On the other hand, increased levels of TnT were associated with weakness and reduced daily living function but not cardiac involvement [229].

### 6.11. Cancer Therapy

#### 6.11.1. Epidemiology

Tn can become elevated in patients receiving various chemotherapy drugs for cancer treatment, most commonly anthracyclines, trastuzumab, immune checkpoint inhibitors, and vascular endothelial growth factor (VEGF) inhibitors. In a large early study of patients receiving chemotherapy, conventional TnI elevations were seen in 30% of patients on anthracyclines and 14% of patients on trastuzumab [230]. A recent large prospective study found hs-TnI elevations in 11.2% of patients on immune checkpoint inhibitors [231]. Data on Tn elevations in patients on VEGF therapy are scarce, despite the known association between VEGF and LV dysfunction [232].

#### 6.11.2. Hypothesized Mechanism

Tn elevations in patients receiving chemotherapy are often reflective of myocardial injury, which can occur through direct toxic effects on the myocardium (anthracyclines), indirect effects that lead to a decline in cardiac function (trastuzumab and VEGF inhibitors), or inflammatory cell infiltration in the myocardium (checkpoint inhibitors) [232].

#### 6.11.3. Prognostic Implications

Tn assays are an important screening tool for the early assessment of chemotherapy-related cardiac injury. For example, multiple studies found that hs-TnT and hs-TnI elevations were predictive of LV dysfunction in patients on anthracyclines and/or trastuzumab [233,234,235,236]. In patients on immune checkpoint inhibitors, elevated conventional Tn was associated with myocarditis and the risk of MACE, including cardiovascular death, cardiogenic shock, cardiac arrest, or complete heart block [237]. Because Tn elevations can be detected prior to echocardiographic changes, the assay can allow clinicians to identify patients who require cardiotoxicity prevention or treatment and to monitor the response to therapy in patients who have developed cardiotoxicity [232,238].

### 6.12. Gastrointestinal Bleed

#### 6.12.1. Epidemiology

Patients presenting with acute gastrointestinal bleeds (GIBs) may have elevations in Tn, an important finding given that cardiovascular-related deaths account for 30% of deaths in patients with acute GIB who survive the initial bleeding episode [239]. The prevalence of conventional TnI elevation in acute GIB is 10–19%, depending on the study. However, these studies did not control for risk factors and comorbid conditions like chronic kidney disease and coronary artery disease, which were more likely to be found in the TnI-positive group [239,240,241].

#### 6.12.2. Hypothesized Mechanism

The mechanism of Tn elevation in GIB is multifactorial and may include decreased oxygen supply due to anemia, increased oxygen demand due to tachycardia, and, in patients who develop sepsis, cytokine release, leading to increased myocyte permeability [241,242]. Another possible mechanism is that patients with elevated hs-TnT may also have subclinically elevated venous filling pressures, which may increase gastrointestinal mucosal congestion and subsequently increase the likelihood of GIB. Additionally, since studies have found associations between hs-TnT and microvascular disease, an increased incidence of microvascular disease may be contributing to fragile, poorly healing, and injury-prone vessels in the gastrointestinal tract [243].

#### 6.12.3. Prognostic Implications

In patients admitted for GIB, elevated conventional Tn levels are associated with higher mortality, though this may be confounded by the higher prevalence of CKD, CAD, HF, and hypertension in this group [239,240,241]. In one study that adjusted for GIB severity and baseline characteristics, elevated Tn was associated with increased long-term mortality but not increased 30-day mortality [242].

## 7. Clinical Situations in Which a Normal or Mildly Elevated Abnormal Troponin Is Not Reassuring

There are several key situations in which clinicians should not be reassured by a normal or mildly elevated Tn. The first is STEMI, as many patients presenting with STEMI do not necessarily have a Tn elevation. In a cohort of 14,061 patients who presented to the emergency department and underwent primary PCI for STEMI, 47.2% had negative admission conventional Tn [244]. In addition, nearly half of STEMI patients presented in the initial window between ischemia onset and documented subsequent Tn elevation. Therefore, a negative Tn might reflect that the patient is presenting in the early hours of the natural history of STEMI, when the beginnings of ischemia have not yet injured enough myocardium to result in a level of serum Tn above the limit of normal [245]. This effect is likely to be significantly attenuated with the use of hs-Tn assays, which feature a much lower limit of quantification and detection and, as previously mentioned, reduce the “troponin-blind” time interval.

Second are high-risk patterns, sometimes referred to as STEMI equivalents, on ECG. These high-risk patterns on ECG can indicate a critical stenosis or occlusion of a coronary artery and should be evaluated with great vigilance, even if Tn is normal or mildly elevated. One such pattern is the T waves of Wellen’s syndrome [246,247]. Type 1 Wellen’s syndrome involves deep symmetric T-wave inversions in at least V2-V3 (though the involvement of V1-V6, aVL, and I is also possible). Type 2 Wellen’s involves positive-then-negative biphasic T waves in these leads. In addition, the ECG must show no precordial Q waves to indicate prior infarct, preserved precordial R-wave progression, and ST segments that are either isoelectric or less than 1 mm elevated. Another high-risk ECG pattern is De Winter’s sign, also known as persistent hyperacute T-wave syndrome. ECG will show tall symmetric T waves and upsloping ST depression > 1 mm deep at the J-point in the precordial leads V1-V6, especially in V3 [248]. In addition, there will also be an upsloping ST elevation in AVR ≥ 1 mm. None of the precordial leads should have ST elevation; the precordial leads should only have an upsloping ST depression. The presence of either of these De Winter’s signs on ECG has a high likelihood of signifying critical, proximal left anterior descending or left main artery stenosis, and there should be a low threshold to pursue coronary angiography regardless of Tn values. 

Third is immune-checkpoint-inhibitor-related (ICI) myocarditis, a rare but dangerous immune-related adverse event caused by ICI therapy. ICIs are therapies that inhibit the negative regulators of the T-cell immune response, including programmed cell death protein-1 (PD-1), PD-1 ligand (PD-L1), and cytotoxic T lymphocyte-associated protein-4 (CTLA-4) [249]. Currently approved ICIs include ipilimumab, nivolumab, pembrolizumab, cemiplimab, atezolizumab, avelumab, and durvalumab. The augmented immune response resulting from the action of ICI can lead to a range of immune-related toxicities, including ICI-related myocarditis. In one case series, ICI myocarditis was associated with a MACE in nearly half (46%) of patients [250], while a large pharmacovigilance study found the incidence of fatality in ICI myocarditis to be 50% [251]. Unfortunately, this condition, with such a high mortality rate, can be difficult to detect. In a recently published case series, 5% of patients with ICI myocarditis had negative hs-Tn values, confounding the diagnosis of the potentially lethal condition [252]. Although it can occur at any time, most cases of ICI myocarditis occur early, with a median time to onset of toxicity of 30 days, which is approximately after the first or second ICI infusion [251]. The receipt of combination ICI therapy (e.g., a CTLA-4 inhibitor combined with a PD-1 inhibitor) is the most well-established risk factor for the development of ICI myocarditis, with the combination of nivolumab and ipilimumab carrying a 4.74-fold risk of myocarditis compared to nivolumab alone [253]. Therefore, in any patient with an oncologic history, it is imperative to ascertain whether there has been recent or current use of ICI agents within the last three years and to maintain a high index of suspicion of the possibility of ICI myocarditis, even if the serum Tn level is low or normal.

## 8. Conclusions

The interpretation of serum Tn elevations in the era of high-sensitivity assays requires a nuanced understanding of a wide array of cardiac and non-cardiac conditions. In this review, we present a comprehensive survey of the prevalence, hypothesized mechanisms, and prognostic implications of Tn elevations in cardiac and non-cardiac conditions. As successive generations of serum Tn assays increase in sensitivity, there is an even greater need for comprehensive contextual interpretation, integrating clinical judgment with laboratory data, to appropriately risk-stratify patients in the diverse landscape of cardiovascular care. Moreover, recognizing situations where a normal or mildly elevated Tn result may not be reassuring is crucial in avoiding misdiagnosis.

## References

[1] Makam A.N., Nguyen O.K. (2015). Use of Cardiac Biomarker Testing in the Emergency Department. JAMA Intern. Med..

[2] Gokhan I., Dong W., Grubman D., Mezue K., Yang D., Wang Y., Gandhi P.U., Kwan J.M., Hu J.-R. Clinical Biochemistry of Serum Troponin. Diagnostics.

[3] Nomenclature and Criteria for Diagnosis of Ischemic Heart Disease (1979). Report of the Joint International Society and Federation of Cardiology/World Health Organization Task Force on Standardization of Clinical Nomenclature. Circulation.

[4] Alpert J.S., Thygesen K., Antman E., Bassand J.P. (2000). Myocardial Infarction Redefined—A Consensus Document of The Joint European Society of Cardiology/American College of Cardiology Committee for the Redefinition of Myocardial Infarction. J. Am. Coll. Cardiol..

[5] Thygesen K., Alpert J.S., Jaffe A.S., Chaitman B.R., Bax J.J., Morrow D.A., White H.D. (2018). Fourth Universal Definition of Myocardial Infarction (2018). Circulation.

[6] Thygesen K., Alpert J.S., Jaffe A.S., Simoons M.L., Chaitman B.R., White H.D., Thygesen K., Alpert J.S., White H.D., Jaffe A.S. (2012). Third Universal Definition of Myocardial Infarction. J. Am. Coll. Cardiol..

[7] Lang W.R., Cahill K.E., Wark T.W., Gardner R.L. (2022). What We Talk about When We Talk About Troponin: A Descriptive Study of Troponin Terms. Brown Hosp. Med..

[8] McCarthy C.P., Raber I., Chapman A.R., Sandoval Y., Apple F.S., Mills N.L., Januzzi J.L. (2019). Myocardial Injury in the Era of High-Sensitivity Cardiac Troponin Assays: A Practical Approach for Clinicians. JAMA Cardiol..

[9] Twerenbold R., Jaffe A., Reichlin T., Reiter M., Mueller C. (2012). High-Sensitive Troponin T Measurements: What Do We Gain and What Are the Challenges?. Eur. Heart J..

[10] Wu A.H.B., Christenson R.H., Greene D.N., Jaffe A.S., Kavsak P.A., Ordonez-Llanos J., Apple F.S. (2018). Clinical Laboratory Practice Recommendations for the Use of Cardiac Troponin in Acute Coronary Syndrome: Expert Opinion from the Academy of the American Association for Clinical Chemistry and the Task Force on Clinical Applications of Cardiac Bio-Markers of the International Federation of Clinical Chemistry and Laboratory Medicine. Clin. Chem..

[11] Jaffe A.S., Moeckel M., Giannitsis E., Huber K., Mair J., Mueller C., Plebani M., Thygesen K., Lindahl B. (2014). In Search for the Holy Grail: Suggestions for Studies to Define Delta Changes to Diagnose or Exclude Acute Myocardial Infarction: A Position Paper from the Study Group on Biomarkers of the Acute Cardiovascular Care Association. Eur. Heart J. Acute Cardiovasc. Care.

[12] Apple F.S., Jaffe A.S., Collinson P., Mockel M., Ordonez-Llanos J., Lindahl B., Hollander J., Plebani M., Than M., Chan M.H.M. (2015). IFCC Educational Materials on Selected Analytical and Clinical Applications of High Sensitivity Cardiac Troponin Assays. Clin. Biochem..

[13] Sandoval Y., Jaffe A.S. (2019). Type 2 Myocardial Infarction: JACC Review Topic of the Week. J. Am. Coll. Cardiol..

[14] Smit M., Coetzee A.R., Lochner A. (2020). The Pathophysiology of Myocardial Ischemia and Perioperative Myocardial Infarction. J. Cardiothorac. Vasc. Anesth..

[15] Knuuti J., Wijns W., Saraste A., Capodanno D., Barbato E., Funck-Brentano C., Prescott E., Storey R.F., Deaton C., Cuisset T. (2020). 2019 ESC Guidelines for the Diagnosis and Management of Chronic Coronary Syndromes. Eur. Heart J..

[16] Virani S.S., Newby L.K., Arnold S.V., Bittner V., Brewer L.C., Demeter S.H., Dixon D.L., Fearon W.F., Hess B., Johnson H.M. (2023). 2023 AHA/ACC/ACCP/ASPC/NLA/PCNA Guideline for the Management of Patients with Chronic Coronary Disease: A Report of the American Heart Association/American College of Cardiology Joint Committee on Clinical Practice Guidelines. Circulation.

[17] Katz D., Gavin M.C. (2019). Stable Ischemic Heart Disease. Ann. Intern. Med..

[18] Mehta P.K., Huang J., Levit R.D., Malas W., Waheed N., Bairey Merz C.N. (2022). Ischemia and No Obstructive Coronary Arteries (INOCA): A Narrative Review. Atherosclerosis.

[19] Arbab-Zadeh A., Nakano M., Virmani R., Fuster V. (2012). Acute Coronary Events. Circulation.

[20] Reimer K.A., Jennings R.B., Tatum A.H. (1983). Pathobiology of Acute Myocardial Ischemia: Metabolic, Functional and Ultrastructural Studies. Am. J. Cardiol..

[21] Bentzon J.F., Otsuka F., Virmani R., Falk E. (2014). Mechanisms of Plaque Formation and Rupture. Circ. Res..

[22] Libby P., Pasterkamp G., Crea F., Jang I.-K. (2019). Reassessing the Mechanisms of Acute Coronary Syndromes. Circ. Res..

[23] Sandoval Y., Apple F.S., Smith S.W. (2018). High-Sensitivity Cardiac Troponin Assays and Unstable Angina. Eur. Heart J. Acute Cardiovasc. Care.

[24] Bergamaschi L., Foà A., Paolisso P., Renzulli M., Angeli F., Fabrizio M., Bartoli L., Armillotta M., Sansonetti A., Amicone S. (2023). Prognostic Role of Early Cardiac Magnetic Resonance in Myocardial Infarction with Nonobstructive Coronary Arteries. JACC Cardiovasc. Imaging.

[25] Garcia-Garcia H.M., McFadden E.P., Farb A., Mehran R., Stone G.W., Spertus J., Onuma Y., Morel M., van Es G.-A., Zuckerman B. (2018). Standardized End Point Definitions for Coronary Intervention Trials: The Academic Research Consortium-2 Consensus Document. Eur. Heart J..

[26] Selvanayagam J.B., Petersen S.E., Francis J.M., Robson M.D., Kardos A., Neubauer S., Taggart D.P. (2004). Effects of Off-Pump Versus On-Pump Coronary Surgery on Reversible and Irreversible Myocardial Injury. Circulation.

[27] Thygesen K., Alpert J.S., White H.D., Jaffe A.S., Apple F.S., Galvani M., Katus H.A., Newby L.K., Ravkilde J., Chaitman B. (2007). Universal Definition of Myocardial Infarction: Kristian Thygesen, Joseph S. Alpert and Harvey D. White on Behalf of the Joint ESC/ACCF/AHA/WHF Task Force for the Redefinition of Myocardial Infarction. Eur. Heart J..

[28] Jaffe A.S., Wu A.H.B. (2012). Troponin Release—Reversible or Irreversible Injury? Should We Care?. Clin. Chem..

[29] White H.D. (2011). Pathobiology of Troponin Elevations: Do Elevations Occur with Myocardial Ischemia as Well as Necrosis?. J. Am. Coll. Cardiol..

[30] Byrne R.A., Rossello X., Coughlan J.J., Barbato E., Berry C., Chieffo A., Claeys M.J., Dan G.-A., Dweck M.R., Galbraith M. (2023). 2023 ESC Guidelines for the Management of Acute Coronary Syndromes: Developed by the Task Force on the Management of Acute Coronary Syndromes of the European Society of Cardiology (ESC). Eur. Heart J..

[31] Collinson P.O., Heung Y.M., Gaze D., Boa F., Senior R., Christenson R., Apple F.S. (2012). Influence of Population Selection on the 99th Percentile Reference Value for Cardiac Troponin Assays. Clin. Chem..

[32] Shah A.S.V., Griffiths M., Lee K.K., McAllister D.A., Hunter A.L., Ferry A.V., Cruikshank A., Reid A., Stoddart M., Strachan F. (2015). High Sensitivity Cardiac Troponin and the Under-Diagnosis of Myocardial Infarction in Women: Prospective Cohort Study. BMJ.

[33] Kimenai D.M., Lindahl B., Jernberg T., Bekers O., Meex S.J.R., Eggers K.M. (2020). Sex-Specific Effects of Implementing a High-Sensitivity Troponin I Assay in Patients with Suspected Acute Coronary Syndrome: Results from SWEDEHEART Registry. Sci. Rep..

[34] Sandoval Y., Apple F.S., Mahler S.A., Body R., Collinson P.O., Jaffe A.S. (2022). High-Sensitivity Cardiac Troponin and the 2021 AHA/ACC/ASE/CHEST/SAEM/SCCT/SCMR Guidelines for the Evaluation and Diagnosis of Acute Chest Pain. Circulation.

[35] Wallace T.W., Abdullah S.M., Drazner M.H., Das S.R., Khera A., McGuire D.K., Wians F., Sabatine M.S., Morrow D.A., de Lemos J.A. (2006). Prevalence and Determinants of Troponin T Elevation in the General Population. Circulation.

[36] Rubin J., Matsushita K., Lazo M., Ballantyne C.M., Nambi V., Hoogeveen R., Sharrett A.R., Blumenthal R.S., Coresh J., Selvin E. (2016). Determinants of Minimal Elevation in High-Sensitivity Cardiac Troponin T in the General Population. Clin. Biochem..

[37] Lloyd-Jones D.M., Hong Y., Labarthe D., Mozaffarian D., Appel L.J., Van Horn L., Greenlund K., Daniels S., Nichol G., Tomaselli G.F. (2010). Defining and Setting National Goals for Cardiovascular Health Promotion and Disease Reduction. Circulation.

[38] Welsh P., Preiss D., Hayward C., Shah A.S.V., McAllister D., Briggs A., Boachie C., McConnachie A., Padmanabhan S., Welsh C. (2019). Cardiac Troponin T and Troponin I in the General Population. Circulation.

[39] Farmakis D., Mueller C., Apple F.S. (2020). High-Sensitivity Cardiac Troponin Assays for Cardiovascular Risk Stratification in the General Population. Eur. Heart J..

[40] Olivetti G., Giordano G., Corradi D., Melissari M., Lagrasta C., Gambert S.R., Anversa P. (1995). Gender Differences and Aging: Effects on the Human Heart. J. Am. Coll. Cardiol..

[41] Myhre P.L., Claggett B., Ballantyne C.M., Selvin E., Røsjø H., Omland T., Solomon S.D., Skali H., Shah A.M. (2019). Association between Circulating Troponin Concentrations, Left Ventricular Systolic and Diastolic Functions, and Incident Heart Failure in Older Adults. JAMA Cardiol..

[42] Klinkenberg L.J.J., van Dijk J.-W., Tan F.E.S., van Loon L.J.C., van Dieijen-Visser M.P., Meex S.J.R. (2014). Circulating Cardiac Troponin T Exhibits a Diurnal Rhythm. J. Am. Coll. Cardiol..

[43] Willeit P., Welsh P., Evans J.D.W., Tschiderer L., Boachie C., Jukema J.W., Ford I., Trompet S., Stott D.J., Kearney P.M. (2017). High-Sensitivity Cardiac Troponin Concentration and Risk of First-Ever Cardiovascular Outcomes in 154,052 Participants. J. Am. Coll. Cardiol..

[44] Blankenberg S., Salomaa V., Makarova N., Ojeda F., Wild P., Lackner K.J., Jørgensen T., Thorand B., Peters A., Nauck M. (2016). Troponin I and Cardiovascular Risk Prediction in the General Population: The BiomarCaRE Consortium. Eur. Heart J..

[45] Costabel J.P., Urdapilleta M., Lambardi F., Campos R., Vergara J.M., Ariznavarreta P., Trivi M. (2016). High-Sensitivity Cardiac Troponin Levels in Supraventricular Tachyarrhythmias. Pacing Clin. Electrophysiol..

[46] Ben Yedder N., Roux J.F., Paredes F.A. (2011). Troponin Elevation in Supraventricular Tachycardia: Primary Dependence on Heart Rate. Can. J. Cardiol..

[47] Chow G.V., Hirsch G.A., Spragg D.D., Cai J.X., Cheng A., Ziegelstein R.C., Marine J.E. (2010). Prognostic Significance of Cardiac Troponin I Levels in Hospitalized Patients Presenting with Supraventricular Tachycardia. Medicine.

[48] Mariathas M., Olechowski B., Mahmoudi M., Curzen N. (2018). High Sensitivity Troponins in Contemporary Cardiology Practice: Are We Turning a Corner?. Expert Rev. Cardiovasc. Ther..

[49] Korff S., Katus H.A., Giannitsis E. (2006). Differential Diagnosis of Elevated Troponins. Heart.

[50] Redfearn D.P., Ratib K., Marshall H.J., Griffith M.J. (2005). Supraventricular Tachycardia Promotes Release of Troponin I in Patients with Normal Coronary Arteries. Int. J. Cardiol..

[51] Turer A.T., Addo T.A., Martin J.L., Sabatine M.S., Lewis G.D., Gerszten R.E., Keeley E.C., Cigarroa J.E., Lange R.A., Hillis L.D. (2011). Myocardial Ischemia Induced by Rapid Atrial Pacing Causes Troponin T Release Detectable by a Highly Sensitive Assay: Insights from a Coronary Sinus Sampling Study. J. Am. Coll. Cardiol..

[52] Jeremias A., Gibson C.M. (2005). Narrative Review: Alternative Causes for Elevated Cardiac Troponin Levels When Acute Coronary Syndromes Are Excluded. Ann. Intern. Med..

[53] Kurt E., AK R., Eke Kurt Ş.Z., Bahadırlı S., Cimilli Öztürk T. (2021). The Correlation of 30- and 90-Day Mortality Rates with Hs-Troponin I Values Measured in Patients Diagnosed with Paroxysmal Supraventricular Tachycardia in Emergency Service. Hong Kong J. Emerg. Med..

[54] Borkovich M., Jeyashanmugaraja G.P., Stawiarski K., McPherson C. (2020). Prognostic utility of cardiac troponin elevation in patients presenting with supraventricular tachycardia. Chest.

[55] Ghersin I., Zahran M., Azzam Z.S., Suleiman M., Bahouth F. (2020). Prognostic Value of Cardiac Troponin Levels in Patients Presenting with Supraventricular Tachycardias. J. Electrocardiol..

[56] Horwich T.B., Patel J., MacLellan W.R., Fonarow G.C. (2003). Cardiac Troponin I Is Associated with Impaired Hemodynamics, Progressive Left Ventricular Dysfunction, and Increased Mortality Rates in Advanced Heart Failure. Circulation.

[57] Vecchia L.L., Mezzena G., Ometto R., Finocchi G., Bedogni F., Soffiati G., Vincenzi M. (1997). Detectable Serum Troponin I in Patients with Heart Failure of Nonmyocardial Ischemic Origin. Am. J. Cardiol..

[58] Vecchia L.L., Mezzena G., Zanolla L., Paccanaro M., Varotto L., Bonanno C., Ometto R. (2000). Cardiac Troponin I as Diagnostic and Prognostic Marker in Severe Heart Failure. J. Heart Lung Transplant..

[59] Missov E., Mair J. (1999). A Novel Biochemical Approach to Congestive Heart Failure: Cardiac Troponin T. Am. Heart J..

[60] Aimo A., Januzzi J.L., Vergaro G., Ripoli A., Latini R., Masson S., Magnoli M., Anand I.S., Cohn J.N., Tavazzi L. (2018). Prognostic Value of High-Sensitivity Troponin T in Chronic Heart Failure. Circulation.

[61] Januzzi J.L., Filippatos G., Nieminen M., Gheorghiade M. (2012). Troponin Elevation in Patients with Heart Failure: On Behalf of the Third Universal Definition of Myocardial Infarction Global Task Force: Heart Failure Section. Eur. Heart J..

[62] Weil B.R., Suzuki G., Young R.F., Iyer V., Canty J.M. (2018). Troponin Release and Reversible Left Ventricular Dysfunction after Transient Pressure Overload. J. Am. Coll. Cardiol..

[63] Feng J., Schaus B.J., Fallavollita J.A., Lee T.-C., Canty J.M. (2001). Preload Induces Troponin I Degradation Independently of Myocardial Ischemia. Circulation.

[64] Cheng W., Li B., Kajstura J., Li P., Wolin M.S., Sonnenblick E.H., Hintze T.H., Olivetti G., Anversa P. (1995). Stretch-Induced Programmed Myocyte Cell Death. J. Clin. Investig..

[65] Hessel M.H.M., Atsma D.E., van der Valk E.J.M., Bax W.H., Schalij M.J., van der Laarse A. (2008). Release of Cardiac Troponin I from Viable Cardiomyocytes Is Mediated by Integrin Stimulation. Pflug. Arch. Eur. J. Physiol..

[66] Konstantinidis K., Whelan R.S., Kitsis R.N. (2012). Mechanisms of Cell Death in Heart Disease. Arterioscler. Thromb. Vasc. Biol..

[67] Logeart D., Beyne P., Cusson C., Tokmakova M., Leban M., Guiti C., Bourgoin P., Solal A.C. (2001). Evidence of Cardiac Myolysis in Severe Nonischemic Heart Failure and the Potential Role of Increased Wall Strain. Am. Heart J..

[68] Park K.C., Gaze D.C., Collinson P.O., Marber M.S. (2017). Cardiac Troponins: From Myocardial Infarction to Chronic Disease. Cardiovasc. Res..

[69] Agewall S., Giannitsis E., Jernberg T., Katus H. (2011). Troponin Elevation in Coronary vs. Non-Coronary Disease. Eur. Heart J..

[70] Miller W.L., Hartman K.A., Burritt M.F., Burnett J.C., Jaffe A.S. (2007). Troponin, B-Type Natriuretic Peptides and Outcomes in Severe Heart Failure: Differences between Ischemic and Dilated Cardiomyopathies. Clin. Cardiol..

[71] Latini R., Masson S., Anand I.S., Missov E., Carlson M., Vago T., Angelici L., Barlera S., Parrinello G., Maggioni A.P. (2007). Prognostic Value of Very Low Plasma Concentrations of Troponin T in Patients with Stable Chronic Heart Failure. Circulation.

[72] Nagarajan V., Hernandez A.V., Tang W.H.W. (2012). Prognostic Value of Cardiac Troponin in Chronic Stable Heart Failure: A Systematic Review. Heart.

[73] Smith S.C., Ladenson J.H., Mason J.W., Jaffe A.S. (1997). Elevations of Cardiac Troponin I Associated with Myocarditis. Circulation.

[74] Lauer B., Niederau C., Kühl U., Schannwell M., Pauschinger M., Strauer B.-E., Schultheiss H.-P. (1997). Cardiac Troponin T in Patients with Clinically Suspected Myocarditis. J. Am. Coll. Cardiol..

[75] Giladi E., Ratzon R., Arow Z., Losin I., Omelchenko A., Pereg D., Assali A., Arnson Y. (2023). Associations between High Sensitivity Troponin in the Emergency Department and Diagnosis of Myocarditis. Austin J. Clin. Cardiol..

[76] Imazio M., Demichelis B., Cecchi E., Belli R., Ghisio A., Bobbio M., Trinchero R. (2003). Cardiac Troponin I in Acute Pericarditis. J. Am. Coll. Cardiol..

[77] Bonnefoy E., Godon P., Kirkorian G., Fatemi M., Chevalier P., Touboul P. (2000). Serum Cardiac Troponin I and ST-Segment Elevation in Patients with Acute Pericarditis. Eur. Heart J..

[78] Yilmaz A., Mahrholdt H., Athanasiadis A., Vogelsberg H., Meinhardt G., Voehringer M., Kispert E.-M., Deluigi C., Baccouche H., Spodarev E. (2008). Coronary Vasospasm as the Underlying Cause for Chest Pain in Patients with PVB19 Myocarditis. Heart.

[79] Massin M., Crochelet A.-S., Jacquemart C. (2017). Acute Myocarditis with Very High Troponin but No Ventricular Dysfunction. Clin. Pediatr..

[80] Chauin A. (2021). The Main Causes and Mechanisms of Increase in Cardiac Troponin Concentrations Other Than Acute Myocardial Infarction (Part 1): Physical Exertion, Inflammatory Heart Disease, Pulmonary Embolism, Renal Failure, Sepsis. VHRM.

[81] Brandt R.R., Filzmaier K., Hanrath P. (2001). Circulating Cardiac Troponin I in Acute Pericarditis. Am. J. Cardiol..

[82] Imazio M., Brucato A., Barbieri A., Ferroni F., Maestroni S., Ligabue G., Chinaglia A., Cumetti D., Della Casa G., Bonomi F. (2013). Good Prognosis for Pericarditis with and without Myocardial Involvement: Results from a Multicenter, Prospective Cohort Study. Circulation.

[83] Purcell J.B., Patel M., Khera A., de Lemos J.A., Forbess L.W., Baker S., Cabell C.H., Peterson G.E. (2008). Relation of Troponin Elevation to Outcome in Patients with Infective Endocarditis. Am. J. Cardiol..

[84] Tsenovoy P., Aronow W.S., Joseph J., Kopacz M.S. (2008). Patients with Infective Endocarditis and Increased Cardiac Troponin I Levels Have a Higher Incidence of In-Hospital Mortality and Valve Replacement than Those with Normal Cardiac Troponin I Levels. Cardiology.

[85] Watkin R.W., Lang S., Smith J.M., Elliott T.S.J., Littler W.A. (2004). Role of Troponin I in Active Infective Endocarditis. Am. J. Cardiol..

[86] Postigo A., Bermejo J., Muñoz P., Valerio M., Marín M., Pinilla B., Cuerpo G., Marí A., Delgado-Montero A., Bouza E. (2020). Troponin Elevation Is Very Common in Patients with Infective Endocarditis and Is Associated with a Poor Outcome. Int. J. Cardiol..

[87] Acosta G., Amro A., Aguilar R., Abusnina W., Bhardwaj N., Koromia G.A., Studeny M., Irfan A., Acosta G., Amro A. (2020). Clinical Determinants of Myocardial Injury, Detectable and Serial Troponin Levels among Patients with Hypertensive Crisis. Cureus.

[88] Gupta K., Kiran M., Chhabra S., Mehta M., Kumar N. (2022). Prevalence, Determinants and Clinical Significance of Cardiac Troponin-I Elevation among Individuals with Hypertensive Emergency: A Prospective Observational Study. Indian J. Crit. Care Med..

[89] Talle M.A., Ngarande E., Doubell A.F., Herbst P.G. (2022). Cardiac Complications of Hypertensive Emergency: Classification, Diagnosis and Management Challenges. J. Cardiovasc. Dev. Dis..

[90] Maheshwarappa H.M., Rai A.V. (2022). Relevance of Troponin I Elevation among Individuals with Hypertensive Emergency. Indian J. Crit. Care Med..

[91] van den Born B.-J.H., Löwenberg E.C., van der Hoeven N.V., de Laat B., Meijers J.C., Levi M., van Montfrans G.A. (2011). Endothelial Dysfunction, Platelet Activation, Thrombogenesis and Fibrinolysis in Patients with Hypertensive Crisis. J. Hypertens..

[92] Olsen F. (1978). Acute Hypertensive Damage of Arterial Vessels of the Heart. Acta Pathol. Microbiol. Scand. Sect. A Pathol..

[93] Kim W., Kim B.S., Kim H.-J., Lee J.H., Shin J., Shin J.-H. (2022). Clinical Implications of Cardiac Troponin-I in Patients with Hypertensive Crisis Visiting the Emergency Department. Ann. Med..

[94] Lee K.K., Noaman A., Vaswani A., Gibbins M., Griffiths M., Chapman A.R., Strachan F., Anand A., McAllister D.A., Newby D.E. (2019). Prevalence, Determinants, and Clinical Associations of High-Sensitivity Cardiac Troponin in Patients Attending Emergency Departments. Am. J. Med..

[95] Pattanshetty D.J., Bhat P.K., Aneja A., Pillai D.P. (2012). Elevated Troponin Predicts Long-Term Adverse Cardiovascular Outcomes in Hypertensive Crisis: A Retrospective Study. J. Hypertens..

[96] Vrsalovic M. (2016). Prognostic Effect of Cardiac Troponin Elevation in Acute Aortic Dissection: A Meta-Analysis. Int. J. Cardiol..

[97] Li G., Wu X.-W., Lu W.-H., Cheng J., Wu X.-Y., Ai R., Zhou Z.-H., Tang Z.-Z., Liao Y.-H. (2016). High-Sensitivity Cardiac Troponin T: A Biomarker for the Early Risk Stratification of Type-A Acute Aortic Dissection?. Arch. Cardiovasc. Dis..

[98] Suzuki T., Distante A., Zizza A., Trimarchi S., Villani M., Salerno Uriarte J.A., de Luca Tupputi Schinosa L., Renzulli A., Sabino F., Nowak R. (2008). Preliminary Experience with the Smooth Muscle Troponin-like Protein, Calponin, as a Novel Biomarker for Diagnosing Acute Aortic Dissection. Eur. Heart J..

[99] Takahashi K., Hiwada K., Kokubu T. (1988). Vascular Smooth Muscle Calponin. A Novel Troponin T-like Protein. Hypertension.

[100] Vagnarelli F., Corsini A., Bugani G., Lorenzini M., Longhi S., Bacchi Reggiani M.L., Biagini E., Graziosi M., Cinti L., Norscini G. (2016). Troponin T Elevation in Acute Aortic Syndromes: Frequency and Impact on Diagnostic Delay and Misdiagnosis. Eur. Heart J. Acute Cardiovasc. Care.

[101] Ramaraj R., Sorrell V.L., Movahed M.R. (2009). Levels of Troponin Release Can Aid in the Early Exclusion of Stress-Induced (Takotsubo) Cardiomyopathy. Exp. Clin. Cardiol..

[102] Lyon A.R., Citro R., Schneider B., Morel O., Ghadri J.R., Templin C., Omerovic E. (2021). Pathophysiology of Takotsubo Syndrome: JACC State-of-the-Art Review. J. Am. Coll. Cardiol..

[103] Sharkey S.W., Windenburg D.C., Lesser J.R., Maron M.S., Hauser R.G., Lesser J.N., Haas T.S., Hodges J.S., Maron B.J. (2010). Natural History and Expansive Clinical Profile of Stress (Tako-Tsubo) Cardiomyopathy. J. Am. Coll. Cardiol..

[104] Topf A., Mirna M., Paar V., Motloch L.J., Grueninger J., Dienhart C., Schulze P.C., Brandt M.C., Larbig R., Hoppe U.C. (2022). The Differential Diagnostic Value of Selected Cardiovascular Biomarkers in Takotsubo Syndrome. Clin. Res. Cardiol..

[105] Prasad A. (2007). Apical Ballooning Syndrome. Circulation.

[106] Wittstein I.S., Thiemann D.R., Lima J.A.C., Baughman K.L., Schulman S.P., Gerstenblith G., Wu K.C., Rade J.J., Bivalacqua T.J., Champion H.C. (2005). Neurohumoral Features of Myocardial Stunning Due to Sudden Emotional Stress. N. Engl. J. Med..

[107] Elesber A., Lerman A., Bybee K.A., Murphy J.G., Barsness G., Singh M., Rihal C.S., Prasad A. (2006). Myocardial Perfusion in Apical Ballooning Syndrome: Correlate of Myocardial Injury. Am. Heart J..

[108] Alashi A., Isaza N., Faulx J., Popovic Z.B., Menon V., Ellis S.G., Faulx M., Kapadia S.R., Griffin B.P., Desai M.Y. (2020). Characteristics and Outcomes of Patients with Takotsubo Syndrome: Incremental Prognostic Value of Baseline Left Ventricular Systolic Function. J. Am. Heart Assoc..

[109] Stahli B.E., Schindler M., Cammann V.L., Szawan K.A., Schweiger V., Niederseer D., Schonberger A., Schonberger M., Koleva I., Mercier J.C. (2022). Cardiac Troponin Elevation and Mortality in Takotsubo Syndrome: New Insights from the International Takotsubo (InterTAK) Registry. Eur. Heart J..

[110] Templin C., Ghadri J.R., Diekmann J., Napp L.C., Bataiosu D.R., Jaguszewski M., Cammann V.L., Sarcon A., Geyer V., Neumann C.A. (2015). Clinical Features and Outcomes of Takotsubo (Stress) Cardiomyopathy. N. Engl. J. Med..

[111] Apridonidze T., Steingart R.M., Comenzo R.L., Hoffman J., Goldsmith Y., Bella J.N., Landau H., Liu J.E. (2012). Clinical and Echocardiographic Correlates of Elevated Troponin in Amyloid Light-Chain Cardiac Amyloidosis. Am. J. Cardiol..

[112] Qian G., Wu C., Zhang Y., Chen Y.-D., Dong W., Ren Y.-H. (2014). Prognostic Value of High-Sensitivity Cardiac Troponin T in Patients with Endomyocardial-Biopsy Proven Cardiac Amyloidosis. J. Geriatr. Cardiol..

[113] Takashio S., Yamamuro M., Izumiya Y., Hirakawa K., Marume K., Yamamoto M., Ueda M., Yamashita T., Ishibashi-Ueda H., Yasuda S. (2018). Diagnostic Utility of Cardiac Troponin T Level in Patients with Cardiac Amyloidosis. ESC Heart Fail..

[114] Kristen A.V., Giannitsis E., Lehrke S., Hegenbart U., Konstandin M., Lindenmaier D., Merkle C., Hardt S., Schnabel P.A., Röcken C. (2010). Assessment of Disease Severity and Outcome in Patients with Systemic Light-Chain Amyloidosis by the High-Sensitivity Troponin T Assay. Blood.

[115] Mueller P.S., Edwards W.D., Gertz M.A. (2000). Symptomatic Ischemic Heart Disease Resulting from Obstructive Intramural Coronary Amyloidosis. Am. J. Med..

[116] Neben-Wittich M.A., Wittich C.M., Mueller P.S., Larson D.R., Gertz M.A., Edwards W.D. (2005). Obstructive Intramural Coronary Amyloidosis and Myocardial Ischemia Are Common in Primary Amyloidosis. Am. J. Med..

[117] Migrino R.Q., Truran S., Gutterman D.D., Franco D.A., Bright M., Schlundt B., Timmons M., Motta A., Phillips S.A., Hari P. (2011). Human Microvascular Dysfunction and Apoptotic Injury Induced by AL Amyloidosis Light Chain Proteins. Am. J. Physiol.-Heart Circ. Physiol..

[118] Modesto K.M., Dispenzieri A., Gertz M., Cauduro S.A., Khandheria B.K., Seward J.B., Kyle R., Wood C.M., Bailey K.R., Tajik A.J. (2007). Vascular Abnormalities in Primary Amyloidosis. Eur. Heart J..

[119] Dispenzieri A., Gertz M.A., Kyle R.A., Lacy M.Q., Burritt M.F., Therneau T.M., McConnell J.P., Litzow M.R., Gastineau D.A., Tefferi A. (2004). Prognostication of Survival Using Cardiac Troponins and N-Terminal pro-Brain Natriuretic Peptide in Patients with Primary Systemic Amyloidosis Undergoing Peripheral Blood Stem Cell Transplantation. Blood.

[120] Labarrere C.A., Nelson D.R., Cox C.J., Pitts D., Kirlin P., Halbrook H. (2000). Cardiac-Specific Troponin I Levels and Risk of Coronary Artery Disease and Graft Failure Following Heart Transplantation. JAMA.

[121] Fitzsimons S.J., Evans J.D.W., Rassl D.M., Lee K.K., Strachan F.E., Parameshwar J., Mills N.L., Pettit S.J. (2022). High-Sensitivity Cardiac Troponin Is Not Associated with Acute Cellular Rejection after Heart Transplantation. Transplantation.

[122] Zimmermann R., Baki S., Dengler T.J., Ring G.H., Remppis A., Lange R., Hagl S., Kübler W., Katus H.A. (1993). Troponin T Release after Heart Transplantation. Heart.

[123] M’Pembele R., Roth S., Nucaro A., Stroda A., Tenge T., Lurati Buse G., Bönner F., Scheiber D., Ballázs C., Tudorache I. (2023). Postoperative High-Sensitivity Troponin T Predicts 1-Year Mortality and Days Alive and out of Hospital after Orthotopic Heart Transplantation. Eur. J. Med. Res..

[124] Patel K., DeStefano R., Yadalam A., Beshiri A., Almuwaqqat Z., Desai S., Book W., Quyyumi A. (2022). High Sensitivity Troponin I as a Predictor of Survival in Heart Transplant Patients. J. Heart Lung Transplant..

[125] Liu Z., Perry L.A., Penny-Dimri J.C., Handscombe M., Overmars I., Plummer M., Segal R., Smith J.A. (2022). Elevated Cardiac Troponin to Detect Acute Cellular Rejection after Cardiac Transplantation: A Systematic Review and Meta-Analysis. Transpl. Int..

[126] Haegeli L.M., Kotschet E., Byrne J., Adam D.C., Lockwood E.E., Leather R.A., Sterns L.D., Novak P.G. (2008). Cardiac Injury after Percutaneous Catheter Ablation for Atrial Fibrillation. EP Eur..

[127] Madrid A.H., del Rey J.M., Rubí J., Ortega J., González Rebollo J.M., Seara J.G., Ripoll E., Moro C. (1998). Biochemical Markers and Cardiac Troponin I Release after Radiofrequency Catheter Ablation: Approach to Size of Necrosis. Am. Heart J..

[128] Reichlin T., Hochholzer W., Bassetti S., Steuer S., Stelzig C., Hartwiger S., Biedert S., Schaub N., Buerge C., Potocki M. (2009). Early Diagnosis of Myocardial Infarction with Sensitive Cardiac Troponin Assays. N. Engl. J. Med..

[129] Lund M., French J.K., Johnson R.N., Williams B.F., White H.D. (2000). Serum Troponins T and I after Elective Cardioversion. Eur. Heart J..

[130] Rao A.C.R., Naeem N., John C., Collinson P.O., Canepa-Anson R., Joseph S.P. (1998). Direct Current Cardioversion Does Not Cause Cardiac Damage: Evidence from Cardiac Troponin T Estimation. Heart.

[131] Allan J.J., Feld R.D., Russell A.A., Ladenson J.H., Rogers M.A.M., Kerber R.E., Jaffe A.S. (1997). Cardiac Troponin I Levels Are Normal or Minimally Elevated after Transthoracic Cardioversion. J. Am. Coll. Cardiol..

[132] Bonnefoy E., Chevalier P., Kirkorian G., Guidolet J., Marchand A., Touboul P. (1997). Cardiac Troponin I Does Not Increase after Cardioversion. Chest.

[133] Piechota W., Gielerak G., Ryczek R., Kaźmierczak A., Bejm J., Piechota W. (2007). Cardiac Troponin I after External Electrical Cardioversion for Atrial Fibrillation as a Marker of Myocardial Injury—A Preliminary Report. Kardiol. Pol..

[134] Lobo R., Jaffe A.S., Cahill C., Blake O., Abbas S., Meany T.B., Hennessy T., Kiernan T.J. (2018). Significance of High-Sensitivity Troponin T after Elective External Direct Current Cardioversion for Atrial Fibrillation or Atrial Flutter. Am. J. Cardiol..

[135] Horjen A.W., Ulimoen S.R., Seljeflot I., Smith P., Arnesen H., Norseth J., Tveit A. (2016). High-Sensitivity Troponin I and Rhythm Outcome after Electrical Cardioversion for Persistent Atrial Fibrillation. Cardiology.

[136] Yoshida K., Yui Y., Kimata A., Koda N., Kato J., Baba M., Misaki M., Abe D., Tokunaga C., Akishima S. (2014). Troponin Elevation after Radiofrequency Catheter Ablation of Atrial Fibrillation: Relevance to AF Substrate, Procedural Outcomes, and Reverse Structural Remodeling. Heart Rhythm.

[137] Ilva T.J., Eskola M.J., Nikus K.C., Voipio-Pulkki L.-M., Lund J., Pulkki K., Mustonen H., Niemelä K.O., Karhunen P.J., Porela P. (2010). The Etiology and Prognostic Significance of Cardiac Troponin I Elevation in Unselected Emergency Department Patients. J. Emerg. Med..

[138] Pruszczyk P., Bochowicz A., Torbicki A., Szulc M., Kurzyna M., Fijałkowska A., Kuch-Wocial A. (2003). Cardiac Troponin T Monitoring Identifies High-Risk Group of Normotensive Patients with Acute Pulmonary Embolism. Chest.

[139] Kline J.A., Hernandez-Nino J., Rose G.A., Norton H.J., Camargo C.A. (2006). Surrogate Markers for Adverse Outcomes in Normotensive Patients with Pulmonary Embolism. Crit. Care Med..

[140] Müller-Bardorff M., Weidtmann B., Giannitsis E., Kurowski V., Katus H.A. (2002). Release Kinetics of Cardiac Troponin T in Survivors of Confirmed Severe Pulmonary Embolism. Clin. Chem..

[141] Bikdeli B., Muriel A., Rodríguez C., González S., Briceño W., Mehdipoor G., Piazza G., Ballaz A., Lippi G., Yusen R.D. (2023). High-Sensitivity vs. Conventional Troponin Cutoffs for Risk Stratification in Patients with Acute Pulmonary Embolism. JAMA Cardiol..

[142] Pareek M., Rosberg V., Kristensen A.M.D., Afzal M., Byrne C., Biering-Sorensen T., Vaduganathan M., Bhatt D.L., Lofgren B., Sorensen R. (2023). High-Sensitivity Troponin-T Concentrations and Long-Term Risk of Death in Patients with Acute Pulmonary Embolism. Eur. Heart J..

[143] Roongsritong C., Warraich I., Bradley C. (2004). Common Causes of Troponin Elevations in the Absence of Acute Myocardial Infarction: Incidence and Clinical Significance. Chest.

[144] Kilinc G., Dogan O.T., Berk S., Epozturk K., Ozsahin S.L., Akkurt I. (2012). Significance of Serum Cardiac Troponin I Levels in Pulmonary Embolism. J. Thorac. Dis..

[145] Giannitsis E., Müller-Bardorff M., Kurowski V., Weidtmann B., Wiegand U., Kampmann M., Katus H.A. (2000). Independent Prognostic Value of Cardiac Troponin T in Patients with Confirmed Pulmonary Embolism. Circulation.

[146] Ozsu S., Abul Y., Orem A., Oztuna F., Bulbul Y., Yaman H., Ozlu T. (2013). Predictive Value of Troponins and Simplified Pulmonary Embolism Severity Index in Patients with Normotensive Pulmonary Embolism. Multidiscip. Respir. Med..

[147] Becattini C., Vedovati M.C., Agnelli G. (2007). Prognostic Value of Troponins in Acute Pulmonary Embolism: A Meta-Analysis. Circulation.

[148] El-Menyar A., Sathian B., Al-Thani H. (2019). Elevated Serum Cardiac Troponin and Mortality in Acute Pulmonary Embolism: Systematic Review and Meta-Analysis. Respir. Med..

[149] Binder L., Pieske B., Olschewski M., Geibel A., Klostermann B., Reiner C., Konstantinides S. (2005). N-Terminal pro-Brain Natriuretic Peptide or Troponin Testing Followed by Echocardiography for Risk Stratification of Acute Pulmonary Embolism. Circulation.

[150] Ebner M., Guddat N., Keller K., Merten M.C., Lerchbaumer M.H., Hasenfuß G., Konstantinides S.V., Lankeit M. (2020). High-Sensitivity Troponin I for Risk Stratification in Normotensive Pulmonary Embolism. ERJ Open Res..

[151] Filusch A., Giannitsis E., Katus H.A., Meyer F.J. (2010). High-Sensitive Troponin T: A Novel Biomarker for Prognosis and Disease Severity in Patients with Pulmonary Arterial Hypertension. Clin. Sci..

[152] Torbicki A., Kurzyna M., Kuca P., Fijałkowska A., Sikora J., Florczyk M., Pruszczyk P., Burakowski J., Wawrzyńska L. (2003). Detectable Serum Cardiac Troponin T as a Marker of Poor Prognosis Among Patients with Chronic Precapillary Pulmonary Hypertension. Circulation.

[153] Heresi G.A., Tang W.H.W., Aytekin M., Hammel J., Hazen S.L., Dweik R.A. (2012). Sensitive Cardiac Troponin I Predicts Poor Outcomes in Pulmonary Arterial Hypertension. Eur. Respir. J..

[154] Xu S.-L., Yang J., Zhang C.-F., Xu S.-Y., Zhao F.-Y., Liu L.-Q., Xie C.-L., Xing X.-Q., Zhu Y. (2019). Serum Cardiac Troponin Elevation Predicts Mortality in Patients with Pulmonary Hypertension: A Meta-Analysis of Eight Cohort Studies. Clin. Respir. J..

[155] Akwe J., Halford B., Kim E., Miller A. (2018). A Review of Cardiac and Non-Cardiac Causes of Troponin Elevation and Clinical Relevance Part II: Non Cardiac Causes. J. Cardiol. Curr. Res..

[156] Metkus T.S., Guallar E., Sokoll L., Morrow D., Tomaselli G., Brower R., Schulman S., Korley F.K. (2017). Prevalence and Prognostic Association of Circulating Troponin in the Acute Respiratory Distress Syndrome. Crit. Care Med..

[157] Kelley W.E., Januzzi J.L., Christenson R.H. (2009). Increases of Cardiac Troponin in Conditions Other than Acute Coronary Syndrome and Heart Failure. Clin. Chem..

[158] Neukamm A.M.C., Høiseth A.D., Hagve T.-A., Søyseth V., Omland T. (2013). High-Sensitivity Cardiac Troponin T Levels Are Increased in Stable COPD. Heart.

[159] Orde M.M. (2013). Raised Troponin Levels in COPD: A Possible Mechanism. Heart.

[160] Brekke P.H., Omland T., Holmedal S.H., Smith P., Søyseth V. (2008). Troponin T Elevation and Long-Term Mortality after Chronic Obstructive Pulmonary Disease Exacerbation. Eur. Respir. J..

[161] Adamson P.D., Anderson J.A., Brook R.D., Calverley P.M.A., Celli B.R., Cowans N.J., Crim C., Dixon I.J., Martinez F.J., Newby D.E. (2018). Cardiac Troponin I and Cardiovascular Risk in Patients with Chronic Obstructive Pulmonary Disease. J. Am. Coll. Cardiol..

[162] Høiseth A.D., Neukamm A., Karlsson B.D., Omland T., Brekke P.H., Søyseth V. (2011). Elevated High-Sensitivity Cardiac Troponin T Is Associated with Increased Mortality after Acute Exacerbation of Chronic Obstructive Pulmonary Disease. Thorax.

[163] Dubin R.F., Li Y., He J., Jaar B.G., Kallem R., Lash J.P., Makos G., Rosas S.E., Soliman E.Z., Townsend R.R. (2013). Predictors of High Sensitivity Cardiac Troponin T in Chronic Kidney Disease Patients: A Cross-Sectional Study in the Chronic Renal Insufficiency Cohort (CRIC). BMC Nephrol..

[164] Freda B.J., Tang W.H.W., Van Lente F., Peacock W.F., Francis G.S. (2002). Cardiac Troponins in Renal Insufficiency: Review and Clinical Implications. J. Am. Coll. Cardiol..

[165] Wan Nur Aimi W.M.Z., Noorazliyana S., Tuan Salwani T.I., Adlin Zafrulan Z., Najib Majdi Y., Noor Azlin Azraini C.S. (2021). Elevation of Highly Sensitive Cardiac Troponin T Among End-Stage Renal Disease Patients without Acute Coronary Syndrome. Malays. J. Med. Sci..

[166] Jain N., Hedayati S.S. (2011). How Should Clinicians Interpret Cardiac Troponin Values in Patients with ESRD?. Semin. Dial..

[167] Fridén V., Starnberg K., Muslimovic A., Ricksten S.-E., Bjurman C., Forsgard N., Wickman A., Hammarsten O. (2017). Clearance of Cardiac Troponin T with and without Kidney Function. Clin. Biochem..

[168] Khan N.A., Hemmelgarn B.R., Tonelli M., Thompson C.R., Levin A. (2005). Prognostic Value of Troponin T and I among Asymptomatic Patients with End-Stage Renal Disease: A Meta-Analysis. Circulation.

[169] Apple F.S., Murakami M.M., Pearce L.A., Herzog C.A. (2002). Predictive Value of Cardiac Troponin I and T for Subsequent Death in End-Stage Renal Disease. Circulation.

[170] Snaedal S., Bárány P., Lund S.H., Qureshi A.R., Heimbürger O., Stenvinkel P., Löwbeer C., Szummer K. (2021). High-Sensitivity Troponins in Dialysis Patients: Variation and Prognostic Value. Clin. Kidney J..

[171] Hickman P.E., McGill D., Potter J.M., Koerbin G., Apple F.S., Talaulikar G. (2015). Multiple Biomarkers Including Cardiac Troponins T and I Measured by High-Sensitivity Assays, as Predictors of Long-Term Mortality in Patients with Chronic Renal Failure Who Underwent Dialysis. Am. J. Cardiol..

[172] Noppakun K., Ratnachina K., Osataphan N., Phrommintikul A., Wongcharoen W. (2022). Prognostic Values of High Sensitivity Cardiac Troponin T and I for Long-Term Mortality in Hemodialysis Patients. Sci. Rep..

[173] Kraus D., von Jeinsen B., Tzikas S., Palapies L., Zeller T., Bickel C., Fette G., Lackner K.J., Drechsler C., Neumann J.T. (2018). Cardiac Troponins for the Diagnosis of Acute Myocardial Infarction in Chronic Kidney Disease. J. Am. Heart Assoc..

[174] Song D., de Zoysa J.R., Ng A., Chiu W. (2012). Troponins in Acute Kidney Injury. Ren. Fail..

[175] Omar A., Sivadasan P., Hanoura S., Sudarsanan S., Shouman Y., Ragab H., Tuli A., Singh R., Al Khulaifi A. (2015). Influence of Acute Kidney Injury on High Sensitive Troponin after Cardiac Surgery. A Single Center Retrospective Observational Study. Intensive Care Med. Exp..

[176] Banerjee D., Perrett C., Banerjee A. (2019). Troponins, Acute Coronary Syndrome and Renal Disease: From Acute Kidney Injury Through End-Stage Kidney Disease. Eur. Cardiol..

[177] Scheitz J.F., Stengl H., Nolte C.H., Landmesser U., Endres M. (2021). Neurological Update: Use of Cardiac Troponin in Patients with Stroke. J. Neurol..

[178] Scheitz J.F., Nolte C.H., Doehner W., Hachinski V., Endres M. (2018). Stroke–Heart Syndrome: Clinical Presentation and Underlying Mechanisms. Lancet Neurol..

[179] Scheitz J.F., Mochmann H.-C., Erdur H., Tütüncü S., Haeusler K.G., Grittner U., Laufs U., Endres M., Nolte C.H. (2014). Prognostic Relevance of Cardiac Troponin T Levels and Their Dynamic Changes Measured with a High-Sensitivity Assay in Acute Ischaemic Stroke: Analyses from the TRELAS Cohort. Int. J. Cardiol..

[180] Faiz K.W., Thommessen B., Einvik G., Omland T., Rønning O.M. (2014). Prognostic Value of High-Sensitivity Cardiac Troponin T in Acute Ischemic Stroke. J. Stroke Cerebrovasc. Dis..

[181] van der Bilt I.a.C., Hasan D., Vandertop W.P., Wilde A.a.M., Algra A., Visser F.C., Rinkel G.J.E. (2009). Impact of Cardiac Complications on Outcome after Aneurysmal Subarachnoid Hemorrhage: A Meta-Analysis. Neurology.

[182] Zhang L., Zhang B., Qi S. (2020). Impact of Echocardiographic Wall Motion Abnormality and Cardiac Biomarker Elevation on Outcome after Subarachnoid Hemorrhage: A Meta-Analysis. Neurosurg. Rev..

[183] Gerner S.T., Auerbeck K., Sprügel M.I., Sembill J.A., Madžar D., Gölitz P., Hoelter P., Kuramatsu J.B., Schwab S., Huttner H.B. (2018). Peak Troponin I Levels Are Associated with Functional Outcome in Intracerebral Hemorrhage. Cerebrovasc. Dis..

[184] Ahn S.-H., Kim Y.-H., Shin C.-H., Lee J.-S., Kim B.-J., Kim Y.-J., Noh S.-M., Kim S.-M., Kang H.-G., Kang D.-W. (2016). Cardiac Vulnerability to Cerebrogenic Stress as a Possible Cause of Troponin Elevation in Stroke. J. Am. Heart Assoc..

[185] Wrigley P., Khoury J., Eckerle B., Alwell K., Moomaw C.J., Woo D., Flaherty M.L., De Los Rios la Rosa F., Mackey J., Adeoye O. (2017). Prevalence of Positive Troponin and Echocardiogram Findings and Association with Mortality in Acute Ischemic Stroke. Stroke.

[186] Sposato L.A., Hilz M.J., Aspberg S., Murthy S.B., Bahit M.C., Hsieh C.-Y., Sheppard M.N., Scheitz J.F. (2020). Post-Stroke Cardiovascular Complications and Neurogenic Cardiac Injury: JACC State-of-the-Art Review. J. Am. Coll. Cardiol..

[187] Zahid T., Eskander N., Emamy M., Ryad R., Jahan N. (2020). Cardiac Troponin Elevation and Outcome in Subarachnoid Hemorrhage. Cureus.

[188] Umeoji K., Yoganathan U., Palathingal A.A. (2022). Elevated Troponin in Patients with Intracerebral Hemorrhage. Adv. Clin. Med. Res. Healthc. Deliv..

[189] Kallmünzer B., Breuer L., Kahl N., Bobinger T., Raaz-Schrauder D., Huttner H.B., Schwab S., Köhrmann M. (2012). Serious Cardiac Arrhythmias after Stroke: Incidence, Time Course, and Predictors—A Systematic, Prospective Analysis. Stroke.

[190] Broersen L.H.A., Siegerink B., Sperber P.S., von Rennenberg R., Piper S.K., Nolte C.H., Heuschmann P.U., Endres M., Scheitz J.F., Liman T.G. (2020). High-Sensitivity Cardiac Troponin T and Cognitive Function in Patients with Ischemic Stroke. Stroke.

[191] He Y., Liu Q., Wang J., Wang D.W., Ding H., Wang W. (2019). Prognostic Value of Elevated Cardiac Troponin I in Patients with Intracerebral Hemorrhage. Clin. Cardiol..

[192] Tummala P., Makhlouf N., Kumar A. (2015). Troponin Elevation in Spontaneous Intracranial Hemorrhage (P3.088). Neurology.

[193] Hussain N. (2013). Elevated Cardiac Troponins in Setting of Systemic Inflammatory Response Syndrome, Sepsis, and Septic Shock. ISRN Cardiol..

[194] Frencken J.F., Donker D.W., Spitoni C., Koster-Brouwer M.E., Soliman I.W., Ong D.S.Y., Horn J., van der Poll T., van Klei W.A., Bonten M.J.M. (2018). Myocardial Injury in Patients with Sepsis and Its Association with Long-Term Outcome. Circ. Cardiovasc. Qual. Outcomes.

[195] Jendoubi A., Jerbi S., Maamar E., Abbess A., Samoud Z., Kanzari L., Boutiba I., Ghedira S., Houissa M. (2019). Prognostic Value of High-Sensitivity Troponin I in Patients with Septic Shock: A Prospective Observational Study. Indian J. Crit. Care Med..

[196] Garcia M.A., Rucci J.M., Thai K.K., Lu Y., Kipnis P., Go A.S., Desai M., Bosch N.A., Martinez A., Clancy H. (2021). Association between Troponin I Levels during Sepsis and Postsepsis Cardiovascular Complications. Am. J. Respir. Crit. Care Med..

[197] Bessière F., Khenifer S., Dubourg J., Durieu I., Lega J.-C. (2013). Prognostic Value of Troponins in Sepsis: A Meta-Analysis. Intensive Care Med..

[198] Vallabhajosyula S., Sakhuja A., Geske J.B., Kumar M., Poterucha J.T., Kashyap R., Kashani K., Jaffe A.S., Jentzer J.C. (2017). Role of Admission Troponin-T and Serial Troponin-T Testing in Predicting Outcomes in Severe Sepsis and Septic Shock. J. Am. Heart Assoc..

[199] Regwan S., Hulten E.A., Martinho S., Slim J., Villines T.C., Mitchell J., Slim A.M. (2010). Marathon Running as a Cause of Troponin Elevation: A Systematic Review and Meta-Analysis. J. Interv. Cardiol..

[200] Richardson A.J., Leckie T., Watkins E.R., Fitzpatrick D., Galloway R., Grimaldi R., Baker P. (2018). Post Marathon Cardiac Troponin T Is Associated with Relative Exercise Intensity. J. Sci. Med. Sport.

[201] Vilela E.M., Bastos J.C.C., Rodrigues R.P., Nunes J.P.L. (2014). High-Sensitivity Troponin after Running—A Systematic Review. Neth. J. Med..

[202] Baker P., Leckie T., Harrington D., Richardson A. (2019). Exercise-Induced Cardiac Troponin Elevation: An Update on the Evidence, Mechanism and Implications. Int. J. Cardiol. Heart Vasc..

[203] Omland T., Aakre K.M. (2019). Cardiac Troponin Increase after Endurance Exercise. Circulation.

[204] Orn S., Melberg T.H., Omland T., Skadberg O., Bjorkavoll-Bergseth M.F., Erevik C.B., Hansen M.W., Auestad B., Bergseth R., Aakre K.M. (2020). Is Cardiac Troponin Elevation Following Strenuous Exercise Clinically Relevant in Healthy Subjects?. Eur. Heart J..

[205] Möhlenkamp S., Leineweber K., Lehmann N., Braun S., Roggenbuck U., Perrey M., Broecker-Preuss M., Budde T., Halle M., Mann K. (2013). Coronary Atherosclerosis Burden, but Not Transient Troponin Elevation, Predicts Long-Term Outcome in Recreational Marathon Runners. Basic Res. Cardiol..

[206] Lanza G.A., Mencarelli E., Melita V., Tota A., Gabrielli M., Sarullo F., Cordischi C., Potenza A., Cardone S., Vita A.D. (2019). Post-Exercise High-Sensitivity Troponin T Levels in Patients with Suspected Unstable Angina. PLoS ONE.

[207] Kokowicz P., Stec S., Flasińska K., Budaj A. (2010). Troponin Release Following Exercise Test in Patients with Stable Angina Pectoris—Risk Factors and Prognostic Significance. Kardiol. Pol. (Pol. Heart J.).

[208] Aengevaeren V.L., Baggish A.L., Chung E.H., George K., Kleiven Ø., Mingels A.M.A., Ørn S., Shave R.E., Thompson P.D., Eijsvogels T.M.H. (2021). Exercise-Induced Cardiac Troponin Elevations: From Underlying Mechanisms to Clinical Relevance. Circulation.

[209] Salim A., Velmahos G.C., Jindal A., Chan L., Vassiliu P., Belzberg H., Asensio J., Demetriades D. (2001). Clinically Significant Blunt Cardiac Trauma: Role of Serum Troponin Levels Combined with Electrocardiographic Findings. J. Trauma Acute Care Surg..

[210] Velmahos G.C., Karaiskakis M., Salim A., Toutouzas K.G., Murray J., Asensio J., Demetriades D. (2003). Normal Electrocardiography and Serum Troponin I Levels Preclude the Presence of Clinically Significant Blunt Cardiac Injury. J. Trauma Acute Care Surg..

[211] Keskpaik T., Starkopf J., Kirsimägi Ü., Mihnovitš V., Lomp A., Raamat E.-M., Saar S., Talving P. (2020). The Role of Elevated High-Sensitivity Cardiac Troponin on Outcomes Following Severe Blunt Chest Trauma. Injury.

[212] Marcolini E.G., Keegan J. (2015). Blunt Cardiac Injury. Emerg. Med. Clin. N. Am..

[213] Yousef R., Carr J.A. (2014). Blunt Cardiac Trauma: A Review of the Current Knowledge and Management. Ann. Thorac. Surg..

[214] Huis in ‘t Veld M.A., Craft C.A., Hood R.E. (2018). Blunt Cardiac Trauma Review. Cardiol. Clin..

[215] Decavèle M., Gault N., Gauss T., Pease S., Moyer J.D., Paugam-Burtz C., Foucrier A. (2018). Cardiac Troponin I as an Early Prognosis Biomarker after Trauma: A Retrospective Cohort Study. Br. J. Anaesth..

[216] Martin M., Mullenix P., Rhee P., Belzberg H., Demetriades D., Salim A. (2005). Troponin Increases in the Critically Injured Patient: Mechanical Trauma or Physiologic Stress?. J. Trauma Acute Care Surg..

[217] Kalbitz M., Pressmar J., Stecher J., Weber B., Weiss M., Schwarz S., Miltner E., Gebhard F., Huber-Lang M. (2017). The Role of Troponin in Blunt Cardiac Injury After Multiple Trauma in Humans. World J. Surg..

[218] Li S.F., Zapata J., Tillem E. (2005). The Prevalence of False-Positive Cardiac Troponin I in ED Patients with Rhabdomyolysis. Am. J. Emerg. Med..

[219] Punukollu G., Gowda R.M., Khan I.A., Mehta N.J., Navarro V., Vasavada B.C., Sacchi T.J. (2004). Elevated Serum Cardiac Troponin I in Rhabdomyolysis. Int. J. Cardiol..

[220] Löfberg M., Tähtelä R., Härkönen M., Somer H. (1995). Myosin Heavy-Chain Fragments and Cardiac Troponins in the Serum in Rhabdomyolysis. Diagnostic Specificity of New Biochemical Markers. Arch. Neurol..

[221] Giger R.D., du Fay de Lavallaz J., Prepoudis A., Stoll T., Lopez-Ayala P., Glarner N., Boeddinghaus J., Puelacher C., Nestelberger T., Mueller C. (2020). Rhabdomyolysis. J. Am. Coll. Cardiol..

[222] Ganta N., Gonzalez H., Olufajo O., Mehrotra P.P. (2020). Abstract 15990: Incidence and Outcomes of Elevated Troponins in Patients with Rhabdomyolysis. Circulation.

[223] Schmid J., Liesinger L., Birner-Gruenberger R., Stojakovic T., Scharnagl H., Dieplinger B., Asslaber M., Radl R., Beer M., Polacin M. (2018). Elevated Cardiac Troponin T in Patients with Skeletal Myopathies. J. Am. Coll. Cardiol..

[224] du Fay de Lavallaz J., Prepoudis A., Wendebourg M.J., Kesenheimer E., Kyburz D., Daikeler T., Haaf P., Wanschitz J., Löscher W.N., Schreiner B. (2022). Skeletal Muscle Disorders: A Noncardiac Source of Cardiac Troponin T. Circulation.

[225] Schmid J., Birner-Gruenberger R., Liesinger L., Stojakovic T., Scharnagl H., Dieplinger B., Asslaber M., Radl R., Polacin M., Beer M. (2017). P2612 Elevated Cardiac Troponin T but Not Troponin I in Patients with Skeletal Muscle Disease. Eur. Heart J..

[226] Chaulin A.M. (2022). False-Positive Causes in Serum Cardiac Troponin Levels. J. Clin. Med. Res..

[227] Giannitsis E., Mueller C., Katus H.A. (2019). Skeletal Myopathies as a Non-Cardiac Cause of Elevations of Cardiac Troponin Concentrations. Diagnosis.

[228] Hughes M., Lilleker J.B., Herrick A.L., Chinoy H. (2015). Cardiac Troponin Testing in Idiopathic Inflammatory Myopathies and Systemic Sclerosis-Spectrum Disorders: Biomarkers to Distinguish between Primary Cardiac Involvement and Low-Grade Skeletal Muscle Disease Activity. Ann. Rheum. Dis..

[229] Zhang H., Chi H., Xie L., Sun Y., Liu H., Cheng X., Ye J., Shi H., Hu Q.-Y., Meng J. (2023). The Use of High-Sensitivity Cardiac Troponin I in Assessing Cardiac Involvement and Disease Prognosis in Idiopathic Inflammatory Myopathy. Adv. Rheumatol..

[230] Cardinale D., Sandri M.T., Colombo A., Colombo N., Boeri M., Lamantia G., Civelli M., Peccatori F., Martinelli G., Fiorentini C. (2004). Prognostic Value of Troponin I in Cardiac Risk Stratification of Cancer Patients Undergoing High-Dose Chemotherapy. Circulation.

[231] Waliany S., Neal J.W., Reddy S., Wakelee H., Shah S.A., Srinivas S., Padda S.K., Fan A.C., Colevas A.D., Wu S.M. (2021). Myocarditis Surveillance with High-Sensitivity Troponin I During Cancer Treatment with Immune Checkpoint Inhibitors. JACC CardioOncol..

[232] Sorodoc V., Sirbu O., Lionte C., Haliga R.E., Stoica A., Ceasovschih A., Petris O.R., Constantin M., Costache I.I., Petris A.O. (2022). The Value of Troponin as a Biomarker of Chemotherapy-Induced Cardiotoxicity. Life.

[233] Michel L., Mincu R.I., Mahabadi A.A., Settelmeier S., Al-Rashid F., Rassaf T., Totzeck M. (2020). Troponins and Brain Natriuretic Peptides for the Prediction of Cardiotoxicity in Cancer Patients: A Meta-Analysis. Eur. J. Heart Fail..

[234] Demissei B.G., Hubbard R.A., Zhang L., Smith A.M., Sheline K., McDonald C., Narayan V., Domchek S.M., DeMichele A., Shah P. (2020). Changes in Cardiovascular Biomarkers with Breast Cancer Therapy and Associations with Cardiac Dysfunction. J. Am. Heart Assoc..

[235] Zardavas D., Suter T.M., Van Veldhuisen D.J., Steinseifer J., Noe J., Lauer S., Al-Sakaff N., Piccart-Gebhart M.J., de Azambuja E. (2017). Role of Troponins I and T and N-Terminal Prohormone of Brain Natriuretic Peptide in Monitoring Cardiac Safety of Patients With Early-Stage Human Epidermal Growth Factor Receptor 2-Positive Breast Cancer Receiving Trastuzumab: A Herceptin Adjuvant Study Cardiac Marker Substudy. J. Clin. Oncol..

[236] Sawaya H., Sebag I.A., Plana J.C., Januzzi J.L., Ky B., Tan T.C., Cohen V., Banchs J., Carver J.R., Wiegers S.E. (2012). Assessment of Echocardiography and Biomarkers for the Extended Prediction of Cardiotoxicity in Patients Treated with Anthracyclines, Taxanes, and Trastuzumab. Circ. Cardiovasc. Imaging.

[237] Ganatra S., Parikh R., Neilan T.G. (2019). Cardiotoxicity of Immune Therapy. Cardiol. Clin..

[238] Lv X., Pan C., Guo H., Chang J., Gao X., Wu X., Zhi X., Ren C., Chen Q., Jiang H. (2023). Early Diagnostic Value of High-Sensitivity Cardiac Troponin T for Cancer Treatment-Related Cardiac Dysfunction: A Meta-Analysis. ESC Heart Fail..

[239] Iqbal U., Siddique O., Jameel A., Anwar H., Chaudhary A. (2017). Prognostic Significance of Elevated Cardiac Troponin in Acute Gastrointestinal Bleeding. Gastroenterol. Res..

[240] Kousa O., Addasi Y., Balakrishna A.M., Pajjuru V.S.K., Bardwell J.K., Walters R.W., Ponamgi S., Alla V.M. (2022). Elevated Troponin in Patients with Acute Gastrointestinal Bleeding: Prevalence, Predictors and Outcomes. Future Cardiol..

[241] Iser D.M., Thompson A.J.V., Sia K.K., Yeomans N.D., Chen R.Y.M. (2008). Prospective Study of Cardiac Troponin I Release in Patients with Upper Gastrointestinal Bleeding. J. Gastroenterol. Hepatol..

[242] Vasile V.C., Babuin L., Rio Perez J.A., Alegria J.R., Song L.M.W.K., Chai H.-S., Afessa B., Jaffe A.S. (2009). Long-Term Prognostic Significance of Elevated Cardiac Troponin Levels in Critically Ill Patients with Acute Gastrointestinal Bleeding. Crit. Care Med..

[243] Mathews L., Ishigami J., Ding N., Hoogeveen R.C., Kucharska-Newton A., Ballantyne C.M., Gottesman R., Selvin E., Matsushita K. (2020). Cardiac Biomarkers and Subsequent Risk of Hospitalization with Bleeding in the Community: Atherosclerosis Risk in Communities Study. J. Am. Heart Assoc..

[244] Wanamaker B.L., Seth M.M., Sukul D., Dixon S.R., Bhatt D.L., Madder R.D., Rumsfeld J.S., Gurm H.S. (2019). Relationship Between Troponin on Presentation and In-Hospital Mortality in Patients with ST-Segment–Elevation Myocardial Infarction Undergoing Primary Percutaneous Coronary Intervention. J. Am. Heart Assoc..

[245] Mahajan V.S., Jarolim P. (2011). How to Interpret Elevated Cardiac Troponin Levels. Circulation.

[246] de Zwaan C., Bär F.W., Janssen J.H.A., Cheriex E.C., Dassen W.R.M., Brugada P., Penn O.C.K.M., Wellens H.J.J. (1989). Angiographic and Clinical Characteristics of Patients with Unstable Angina Showing an ECG Pattern Indicating Critical Narrowing of the Proximal LAD Coronary Artery. Am. Heart J..

[247] Zhou L., Gong X., Chen H., Dong T., Cui H., Li H. (2023). Characteristics of Wellens’ Syndrome in the Current PCI Era: A Single-Center Retrospective Study. Emerg. Med. Int..

[248] Morris N.P., Body R. (2017). The De Winter ECG Pattern: Morphology and Accuracy for Diagnosing Acute Coronary Occlusion: Systematic Review. Eur. J. Emerg. Med..

[249] Hu J.-R., Florido R., Lipson E.J., Naidoo J., Ardehali R., Tocchetti C.G., Lyon A.R., Padera R.F., Johnson D.B., Moslehi J. (2019). Cardiovascular Toxicities Associated with Immune Checkpoint Inhibitors. Cardiovasc. Res..

[250] Mahmood S.S., Fradley M.G., Cohen J.V., Nohria A., Reynolds K.L., Heinzerling L.M., Sullivan R.J., Damrongwatanasuk R., Chen C.L., Gupta D. (2018). Myocarditis in Patients Treated with Immune Checkpoint Inhibitors. J. Am. Coll. Cardiol..

[251] Salem J.-E., Manouchehri A., Moey M., Lebrun-Vignes B., Bastarache L., Pariente A., Gobert A., Spano J.-P., Balko J.M., Bonaca M.P. (2018). Cardiovascular Toxicities Associated with Immune Checkpoint Inhibitors: An Observational, Retrospective, Pharmacovigilance Study. Lancet Oncol..

[252] Vasbinder A., Chen Y., Procureur A., Gradone A., Azam T.U., Perry D., Shadid H., Anderson E., Catalan T., Blakely P. (2022). Biomarker Trends, Incidence, and Outcomes of Immune Checkpoint Inhibitor-Induced Myocarditis. JACC CardioOncol..

[253] Johnson D.B., Balko J.M., Compton M.L., Chalkias S., Gorham J., Xu Y., Hicks M., Puzanov I., Alexander M.R., Bloomer T.L. (2016). Fulminant Myocarditis with Combination Immune Checkpoint Blockade. N. Engl. J. Med..

